# Natural Resistance to Ovarian Hyperstimulation Syndrome in Estrildid Finches Reveals Macrophage GPR183 as a Potential Therapeutic Target

**DOI:** 10.1002/advs.75523

**Published:** 2026-05-05

**Authors:** Xiaofei Yan, Yongjie Huang, Jiabao Yang, Su Ma, Songsong Liu, Xuan Huang, Juergen Brosius, Huaping Zheng, Bing Yao, Li Chen, Shanshan Lai, Cheng Deng

**Affiliations:** ^1^ Jiangsu Key Laboratory for Biodiversity and Biotechnology College of Life Sciences Nanjing Normal University Nanjing P. R. China; ^2^ Department of High Altitude Medicine Center for High Altitude Medicine Collage of Life Sciences National Clinical Research Center for Geriatrics West China Hospital Sichuan University Chengdu Sichuan P. R. China; ^3^ Institute of High Altitude Medicine High Altitude Medicine Key Laboratory of Sichuan Province West China Hospital Sichuan University Chengdu Sichuan P. R. China; ^4^ Center of Reproductive Medicine Nanjing Jinling Hospital Clinical School of Medical College Nanjing University Nanjing Jiangsu P. R. China; ^5^ Center For High Altitude Medicine Xining Qinghai P. R. China

**Keywords:** estrildidae, evolutionary medicine, GPR183, macrophage, OHSS, physiological adaptation, scRNA‐seq

## Abstract

Spontaneous ovarian hyperstimulation syndrome (OHSS) is closely associated with follicle stimulating hormone receptor (FSHR) functional mutations. We observed that estrildid finches naturally carry the gain‐of‐function FSHR p.Thr449Ala mutation found in humans, yet do not develop OHSS, thereby providing a novel and system to study aspects of OHSS prevention. Cross‐species single‐cell analysis revealed that macrophages, the most abundant immune cells in ovaries, play a pivotal role in OHSS progression. Macrophage depletion exacerbates the manifestations of OHSS in both birds and rats. Pharmacological activation of the G protein‐coupled receptor 183 (GPR183) in ovarian macrophages, significantly alleviates OHSS symptoms. Mechanistically, GPR183 activation in macrophages maintains ovarian immune homeostasis by downregulating inflammatory factors (Interleukin 1 alpha: IL1A, Interleukin 6: IL6, Interleukin 1 beta: IL1B) and upregulating immune regulators responsive to external stimuli (sphingomyelin phosphodiesterase acid like 3A: *Smpdl3a*, Macrophage‐expressed gene 1: *Mpeg1*, Epithelial stromal interaction 1: *Epsti1*, Unc‐93 homolog B1: *Unc93b1*, Apolipoprotein B mRNA editing enzyme catalytic subunit 1: *Apobec1*). It markedly altered CD44 molecule (*CD44*)/Syndecan‐4 (*SDC4*) ‐mediated intercellular communication between macrophages and endothelial/stromal cells, thereby modulating the ovarian microenvironment. This study identifies ovarian macrophages as a key therapeutic target for OHSS and proposes GPR183 as a novel receptor target for precision macrophage‐based interventions.

## Graphical abstract

Ovarian macrophage depletion reverses OHSS resistance in estrildid finches and exacerbates OHSS symptoms in rats. Activating macrophage GPR183 alleviates OHSS by reducing pro‐inflammatory factors, increasing immunomodulatory molecules, remodeling CD44/SDC4‐mediated communication, and restoring immune homeostasis. This positions macrophages and GPR183 as promising therapeutic targets.

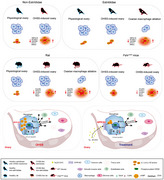

## 1 Introduction

Ovarian hyperstimulation syndrome (OHSS) is a complication resulting from excessive hormonal stimulation of the ovaries and is mainly categorized into iatrogenic OHSS and spontaneous OHSS [1]. The former primarily occurs when infertile women undergo ovarian stimulation with exogenous gonadotropins or follicle‐stimulating hormone (FSH) during assisted reproductive technology, followed by ovulation induction using medications such as human chorionic gonadotropin (hCG) or human menopausal gonadotropin (HMG) [2]. Approximately 20%–30% of controlled ovarian stimulation (COS) patients are susceptible to OHSS [3, 4]. Spontaneous OHSS has been associated with conditions such as molar or multiple pregnancies, cross‐reactivity of follicle stimulating hormone receptor (FSHR) with elevated hCG and thyroid‐stimulating hormone (TSH) in hypothyroidism, as well as activating mutations in the FSHR leading to hypersensitivity to circulating FSH [5, 6]. OHSS patients typically exhibit excessive follicular growth accompanied by symptoms including ovarian enlargement, plasma extravasation, and ascites, severe cases potentially being life‐threatening [7, 8]. To date, several risk factors have been associated with OHSS occurrence. Among these, vascular endothelial growth factor (VEGF) serves as the primary factor promoting increased vascular permeability in OHSS patients [9, 10, 11]. Inhibiting the increase in serum estradiol (E2) levels can prevent the occurrence of OHSS [12].

Recent studies have found that cellular immune cell population activity significantly associated with OHSS risk [13]. Immune activation and cytokine release are important pathogenic factors in the development of OHSS [14]. Macrophages represent a substantial proportion of the most abundant immune cell population in the human, rat, and mouse ovary [15]. They orchestrate regulated inflammatory responses during both physiological ovulation and pathological ovarian tissue injury [15], while also supporting key ovarian functions such as angiogenesis, vascular remodeling [16], follicular development [17], and progesterone‐mediated luteal regulation [18]. Dysfunction of ovarian macrophages is closely associated with local inflammatory responses and the formation of an abnormal biochemical microenvironment, contributing to pathogenesis of various diseases, including ovarian cancer [19, 20], ovarian aging [21, 22], metabolic disorders [23, 24], and polycystic ovary syndrome (PCOS) [25, 26]. Ovarian macrophages sense local signals and modulate physiological processes [27]. For example, pyroptotic macrophage accumulation exacerbates ovarian inflammation through interleukin 1 beta (*Il1b*) and epiregulin secretion and crosstalk with T cells and *EGFR^+^* stromal cells [28]. These findings highlight the important role of immune cells in both physiological and pathological ovarian processes. Nevertheless, their significance in OHSS pathology remains hazy.

In medical intervention, mild to moderate OHSS is typically managed conservatively, while severe cases require prompt hospitalization and active intervention [29]. Current clinical management strategies for OHSS include correction of circulatory volume and electrolyte imbalances, electrolyte replacement, and administration of antibiotics, diuretics, or indomethacin [30]. Studies have also shown that the dopamine receptor agonist cabergoline can significantly reduce the incidence of OHSS, especially early‐onset OHSS, during in vitro fertilization (IVF) and intracytoplasmic sperm injection (ICSI) cycles without affecting pregnancy outcomes [31]. However, treatment options for established OHSS remain limited, and spontaneous OHSS often lacks effective management options. The follicle‐stimulating hormone receptor FSHR, a class A G protein‐coupled receptor (GPCR), normally binds follicle‐stimulating hormone (FSH) to support fertility, folliculogenesis, and recruitment of new antral follicles [32]. Altered FSHR function can cause a spectrum of reproductive disorders. Specifically, gain‐of‐function mutations (e.g., Thr449Ala) heighten receptor sensitivity and predispose individuals to OHSS, while loss‐of‐function mutations cause infertility or primary ovarian insufficiency [33]. Although several pharmacological agents have shown partial efficacy in alleviating OHSS, developing novel GPCR‐targeted therapeutics remains a critical unmet need [34, 35].

Here, we found that female estrildid birds naturally carrying the FSHR p.Thr449Ala variant (a human allele associated with high risk of OHSS) did not develop OHSS symptoms even after ovarian hyperstimulation. The underlying mechanism may involve transcriptional remodeling of ovarian macrophages, which shapes a low‐inflammatory microenvironment, thereby conferring an evolutionarily acquired natural resistance to OHSS. Further investigations using a rat model demonstrated that activation of G protein‐coupled receptor (GPR183) alleviates OHSS symptoms and remodels the ovarian microenvironment by regulating the PKA‐CREB signaling pathway in ovarian macrophages, rebalancing inflammatory and immune response factors, and improving intercellular communication via syndecan‐4 (SDC4) and CD44 molecule (CD44). Together, these findings not only reveal a unique evolutionary adaptive strategy in estrildid birds but also offer potential therapeutic targets and an evolutionary medicine perspective for the intervention of human OHSS.

## 2 Results

### 2.1 Macrophage‐Mediated Anti‐Inflammatory Activity Plays a Critical Role in Underpinning the Resistance to OHSS in Estrildid Finches

**FIGURE 1:**
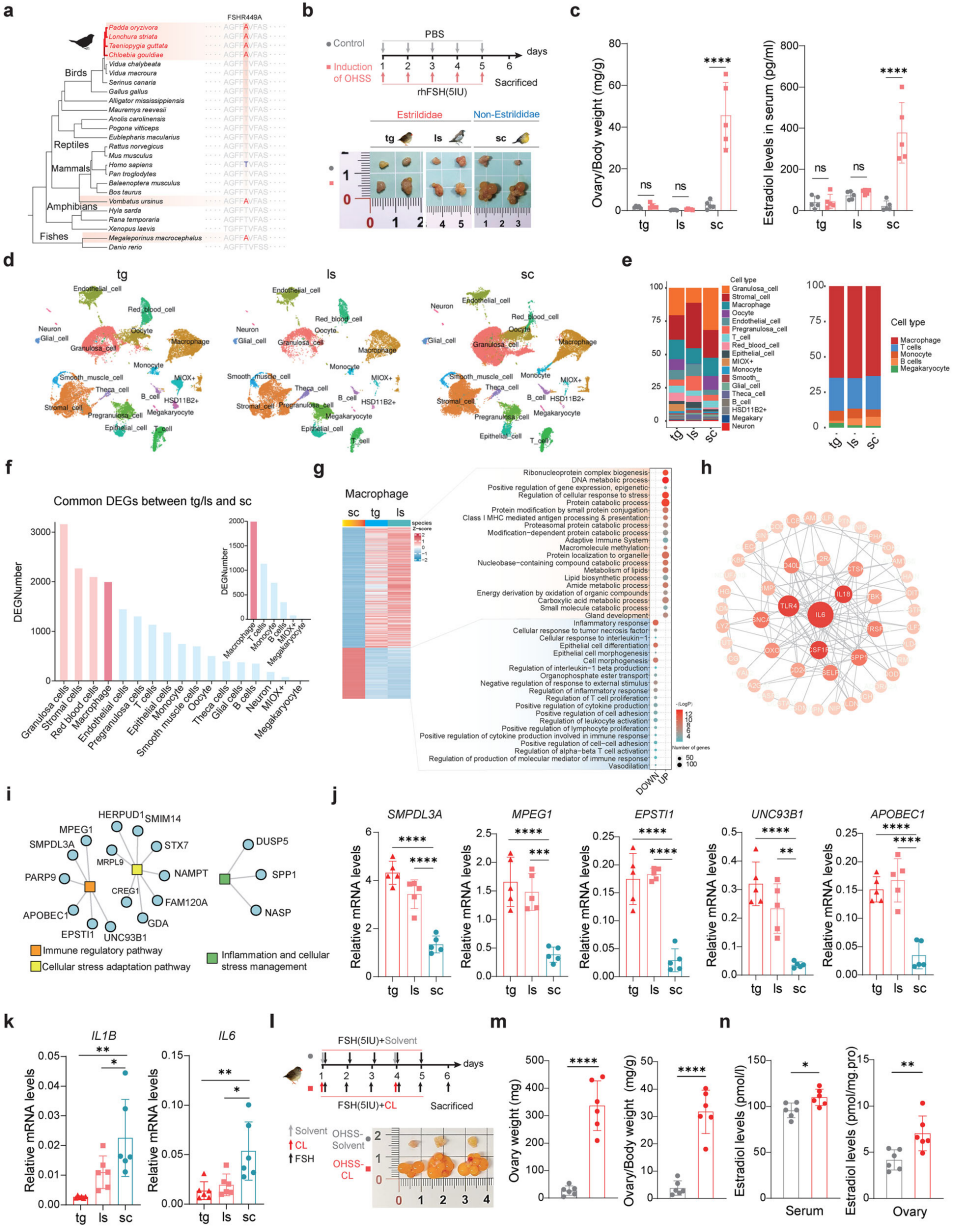
Macrophages confer OHSS resistance via adaptive evolution in estrildid finches (a), We retrieved 3088 vertebrate *FSHR* gene sequences from the NCBI database and conducted online BLAST analysis. Representative chordates were selected to construct a phylogenetic tree using TimeTree, and the tree was visualized with iTOL (https://itol.embl.de/). Species carrying the p.Thr449Ala amino acid substitution are highlighted in red. (b), The induction scheme for an adult bird OHSS model, where the OHSS group received intraperitoneal injections of 5 IU rhFSH for 5 consecutive days. The control group received intraperitoneal injections of an equivalent dose of rhFSH on 3 and 5 days. Estrildid species (zebra finches: tg; Bengalese finches: ls, red, *n* = 5/group) and non‐estrildid species (common canaries: sc, blue, *n* = 5/group) were used. Representative ovary images are shown underneath. (c), Ovary‐to‐body weight ratio and serum estradiol levels for control and OHSS groups in tg, ls, and sc birds (*n* = 5/group). (d), Single‐cell UMAP visualization atlas of ovaries from sexually mature tg, ls, and sc under normal physiological conditions (tg, *n* = 3; ls, *n* = 2; sc, *n* = 3). Each dot represents a cell, colored by cell type. (e), Complete ovarian cell types from the single‐cell analysis of the three bird species (left), and proportions of immune cell subtypes (right). (f), Bar plots displaying the number of shared DEGs across different cell types when comparing estrildid and non‐estrildid species (integrated tg/ls vs. sc), and the number of DEGs in immune cell subtypes (top). (g), Pathway enrichment of differentially expressed genes in macrophages from the tg/ls vs. sc comparison. (h), Protein interaction network for estrildid‐downregulated inflammation pathway‐related enriched DEGs predicted with STRING database. (i), Pathway‐gene network for genes specifically expressed in macrophages predicted using the GeneAgent website, where squares represented pathways and circles represented associated genes. (j), Expression of immune regulation‐related genes in macrophages using qRT‐PCR. (k), Quantitative expression of inflammatory factors *IL1B* and *IL6* in the ovaries of tg, ls, and sc using qRT‐PCR. (l), Scheme for ovarian macrophage depletion in zebra finches using clodronate liposomes (CL) and representative ovary images. Macrophage‐ablated OHSS group (OHSS‐CL) received intraperitoneal injections of 5IU FSH for 5 consecutive days, concurrent with intraperitoneal injections of 30 mg/kg CL on 1 and 3 days. The OHSS group (OHSS‐Solvent) received an equivalent volume of control solvent (*n* = 6). (m,n), Ovary weight, ovary‐to‐body weight ratio (m), and serum and tissue estradiol levels (n) compared between the OHSS‐CL and OHSS‐Solvent groups. The silhouette in panel (a) is adapted from phylopic.org. The images of the tg, ls and sc birds were adapted from the works of Peter Grima, Krayker and Juan Emilio, respectively. All were originally published on Flickr under a CC BY‐SA 2.0 license. tg bird: https://www.flickr.com/photos/wwwpgflickrcom/1744854306/; ls bird: https://www.flickr.com/photos/krayker/2124195933/in/set‐72157603490624994/; sc bird: https://www.flickr.com/photos/juan_e/6942041699/. Data in panel (c) were analyzed using two‐way ANOVA; data in panel (j–k) were analyzed using one‐way ANOVA; data in panels (m,n) were analyzed using unpaired t‐tests. Results were presented as mean ± SEM. Statistically significant differences between experimental and control groups were indicated by ANOVA: ^****^ *p* < 0.0001; ^***^ *p* < 0.001; ^**^ *p* < 0.01; ^*^ *p* < 0.05; ns: *p* >0.05.

Based on the observation that the human p.Thr449Ala variant (*FSHR^449A^*) is typically associated with spontaneous OHSS, we investigated whether this variation occurs in other species. We analyzed FSHR coding sequences from over 3088 vertebrate species available in the NCBI database. The results revealed that the *FSHR* ^449A^ variant naturally occurs in estrildid finches (including *Taeniopygia guttata*, tg; *Lonchura striata domestica*, ls; *Chloebia gouldiae*, cg; and *Padda oryzivora*, po), a marsupial mammal *Vombatus ursinus*, and the fish *Megaleporinus macrocephalus* (Figure 1a; Table S3). We first utilized the CRE‐luciferase reporter assay to verify in vitro whether the *FSHR^449A^* carried by estrildid finches enhances downstream signaling. Upon exogenous stimulation with its ligand rhFSH, we observed that at the same dosage, the naturally occurring *FSHR^449A^* in estrildid finches (tg, ls) exhibited enhanced ligand sensitivity. A similar result was observed with the non‐estrildid finches (*Serinus canaria*, sc, belonging to the family Fringillidae) receptor following introduction of the *FSHR^449A^* variation (Figure S1c). In vivo assessments were then conducted to evaluate the susceptibility of estrildid finches (tg, ls) and non‐estrildid finches (sc) to OHSS. We used rhFSH for induction (Figure 1b). In vitro experiments confirmed that rhFSH can significantly activate tgFSHR, lsFSHR, and scFSHR (Figure S1d). The results showed that rhFSH treatment significantly increased ovary size, ovary‐to‐body weight ratio, serum estradiol levels, and ovarian VEGF expression in non‐estrildid finches, leading to OHSS‐like symptoms. In contrast, these changes were less pronounced in estrildid finches (Figure 1c; Figure S3b). These findings suggest that estrildid finches may have evolved adaptive mechanisms to counteract OHSS susceptibility associated with the *FSHR^449A^*.

To investigate ovarian physiological adaptations, we constructed a multi‐species single‐cell RNA sequencing (scRNA‐seq) of ovarian tissues from two estrildid (tg, ls) and one non‐estrildid (sc) finches. All high‐quality cells were obtained for subsequent analysis (Figure 1d). Based on the expression of canonical marker genes, these clusters were annotated into 18 major homologous cell types: granulosa cells, stromal cells, macrophages, oocytes, endothelial cells, pregranulosa cells, T cells, red blood cells, epithelial cells, *MIOX* ^+^ cells, monocytes, smooth muscle cells, glial cells, theca cells, B cells, *HSD11B2* ^+^ cells, megakaryocytes and neurons (Figure S2a,b). The proportional distribution of cell types was generally consistent across the three species, with macrophages being the most abundant immune cell population in the ovary (Figure 1e).

Based on the ovary's high inflammatory sensitivity and the extensive role of local immunity in ovarian health and disease and follicular remodeling [36, 37], we compared the immune cells in the ovaries of estrildid finches and non‐estrildid finches. Differential expression analysis revealed that macrophages exhibited the most prominent transcriptomic differences within the ovarian immune population of estrildid finches, with 1991 differentially expressed genes (DEGs) identified (Figure 1f). Pathway enrichment analysis indicated that genes with low expression abundance in estrildid finches were enriched in inflammation‐related pathways (e.g., inflammatory response, immune cell activation), while genes with high expression abundance were primarily enriched in pathways related to DNA metabolic processes and the regulation of cellular stress response (Figure 1g). Protein‐protein interaction network analysis of 65 gene products enriched in the inflammatory response pathway identified the pro‐inflammatory cytokine interleukin 6 (*IL6)* as a key regulator (Figure 1h). Furthermore, GeneAgent [38] analysis revealed that highly expressed gene sets in macrophages of female estrildid finches were significantly enriched in pathways related to immune regulation and cellular stress adaptation, while lowly expressed genes were primarily enriched in inflammatory response and cellular stress management pathways (Figure 1i). Based on the scRNA‐seq results, we further validated the expression patterns of the immune regulatory and inflammation‐related genes identified by PPI and GeneAgent analysis (Figure 1h,i) using quantitative real‐time PCR (qRT‐PCR). The results showed that the expression of immune regulatory genes (sphingomyelin phosphodiesterase acid like 3A, *SMPDL3A*; macrophage expressed 1, *MPEG1*; epithelial stromal interaction 1, *EPSTI1*; unc‐93 homolog B1, *UNC93B1*; apolipoprotein B mRNA editing enzyme catalytic subunit 1, *APOBEC1*) was significantly upregulated in estrildid finches (Figure 1j). Compared to non‐estrildid finches (sc), the expression levels of inflammation‐related factors (*IL1B, IL6*) were significantly reduced in the ovarian tissues of estrildid finches (Figure 1k). ELISA results confirmed this finding (Figure S3a). These results suggest that the ovarian microenvironment in estrildid finches may maintain a low‐inflammatory immune state, enabling it to counteract external environmental stimuli more effectively and affect ovarian function.

To investigate whether macrophages are the key cell population responsible for OHSS resistance in estrildid finches, we depleted ovarian macrophages in tg (an estrildid finch) using clodronate liposomes (clodrolip, CL) while establishing an OHSS model (Figure 1l). Effective macrophage depletion was confirmed in vivo by qRT‐PCR analysis of macrophage‐specific markers (complement C1q A chain, *C1QA*; complement C1QB chain, *C1QB*; mannose receptor C‐type 1, *MRC1*) (Figure S3c). The results demonstrated that compared to tg birds pre‐treated with solvent, those pre‐treated with clodrolip exhibited significant increases in ovary weight, ovarian‐to‐body weight ratio, and estradiol levels in both ovarian tissue and serum (Figure 1l–n). Notably, macrophage ablation led to significant downregulation of immune regulatory genes and marked upregulation of inflammatory factors (Figure S3d,e), indicating that the inflammatory response driven by macrophage depletion markedly reversed the inherent OHSS‐resistant phenotype in tg birds.

To further decipher the role of macrophages, we performed bulk RNA‐seq analysis on these OHSS‐like ovaries following macrophage depletion. We observed a broad downregulation of immunomodulatory genes in the OHSS‐like ovaries of tg (Figure S4a,b). The results revealed that upregulated genes were primarily enriched in pathways such as extracellular matrix remodeling, blood vessel development and morphogenesis, and immune cell activation. In contrast, downregulated genes were significantly associated with the regulation of cell cycle, adaptive immune response, and autophagy (Figure S4c). These findings suggest that macrophages are the pivotal cell population conferring OHSS resistance in female zebra finch (*Taeniopygia guttata*, tg), a mechanism potentially mediated through the upregulation of specific immunoregulatory genes, including *SMPDL3A, MPEG1, EPSTI1, UNC93B1*, and *APOBEC1*, to counteract OHSS pathology. This underscores the critical role of macrophages in maintaining ovarian immune homeostasis.

### 2.2 Macrophage Depletion Contributes to the Pathogenesis of OHSS in Rodents

**FIGURE 2:**
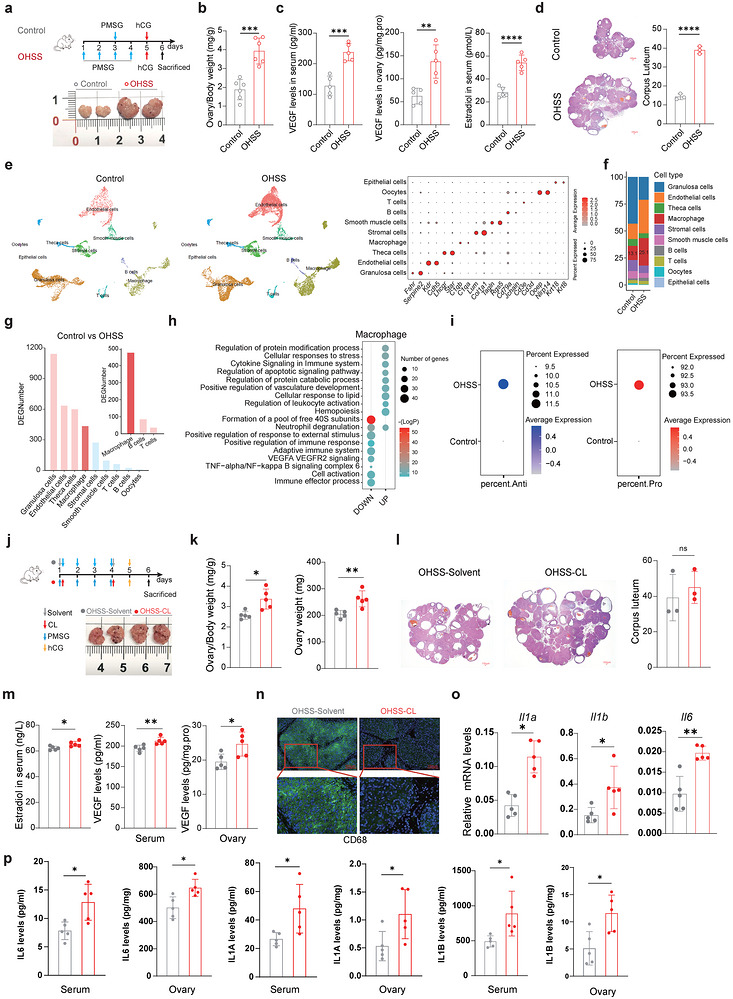
Ovarian macrophages regulate ovarian immune microenvironment during OHSS progression (a), Induction scheme for an OHSS model in Wistar rats and representative ovary images. The OHSS group received intraperitoneal injections of 50 IU PMSG for 4 consecutive days, followed by 30 IU hCG on the fifth day. The control group received intraperitoneal injections of 10 IU PMSG on day 3 and 10 IU hCG on day 5. Ovaries and serum were collected 24 h after hCG administration (*n* = 6). (b), Ovary weight for the control and OHSS groups. (c), Serum and ovarian VEGF levels, as well as serum estradiol levels, in the control group and the OHSS group. (d), Representative H&E staining images of the maximal ovarian tissue section for each individual in the control and OHSS groups (left) and the number of *corpora lutea* (right). *Corpora lutea* in the central area (containing blood clots due to hemorrhage during ovulation) and peripheral area (luteal cells arranged in cord‐like structures, rich in capillaries and connective tissue). Scale bar is 100 µm. (e), UMAP visualization of the single‐cell transcriptome from rat ovaries in control and OHSS groups (Control, *n* = 2; OHSS, *n* = 2). Each dot represented a cell, colored by cell type. Dot plot of cell type‐specific markers for the various cell types in the rat ovary single‐cell data. (f), Proportions of different cell types identified in control and OHSS groups; different colors represent distinct cell populations. (g), Bar graphs depicting the number of DEGs in various cell types from the ovarian single‐cell transcriptomes of control and OHSS groups. Number of DEGs in immune cell subtypes (top). (h), Pathway enrichment analysis of differentially expressed genes in the ovarian macrophage population from control vs. OHSS comparison. (i), Expression ratio of pro‐inflammatory‐related genes (red) and anti‐inflammatory‐related genes (blue) in control vs. OHSS groups. Scale bar = 100 µm. (j,k), Scheme for ovarian macrophage depletion in rats using CL and representative ovary images. Macrophage‐ablated OHSS group (OHSS‐CL) received intraperitoneal injections of 50 IU PMSG for 4 consecutive days and 30 IU hCG on the fifth day, concurrent with intraperitoneal injections of 30 mg/kg CL on days 1 and 3. The OHSS group (OHSS‐Solvent) received an equivalent volume of control solvent (*n* = 6). Ovaries and serum were collected 24 h after hCG administration. Ovary weight and ovary‐to‐body weight ratio for the OHSS‐CL and OHSS‐Solvent groups (k). (l), Representative H&E staining images of the maximal ovarian tissue section for each individual in the OHSS‐CL and OHSS‐Solvent groups (left) and the number of *corpora lutea* (right). (m), Serum and tissue levels of estradiol (left) and VEGF (right) in the OHSS‐CL and OHSS‐Solvent groups. (n), CD68 immunofluorescence staining of ovaries from the OHSS‐CL and OHSS‐Solvent groups. Scale bar = 100 µm. (o), mRNA expression levels of inflammation‐related genes (*Il1a, Il1b, Il6*) as shown by qRT‐PCR. (p), The protein expression levels of inflammation‐related factors (IL1A, IL1B, and IL6) in serum and ovarian tissues were determined by ELISA. Data in panels (b–d, k–m, o,p) were analyzed using unpaired t‐tests. Results were presented as mean ± SEM. Statistically significant differences between experimental and control groups were indicated as follows: ^****^ *p* < 0.0001; ^***^ *p* < 0.001; ^**^ *p* < 0.01; ^*^ *p* < 0.05; ns: *p* >0.05.

To further investigate the role of ovarian macrophages in the pathogenesis of OHSS, we established a rat OHSS model using PMSG and hCG for induction (Figure 2a). Compared with the control group, OHSS model rats exhibited a significant increase in ovarian size, elevated VEGF and estradiol levels (Figure 2a–c), and a marked increase in the number of *corpora lutea* observed in ovarian HE‐stained sections (Figure 2d), confirming successful model establishment.

We then performed scRNA‐seq on fresh unilateral ovarian tissues from both control and OHSS rats (Figure 2e). Using marker gene expression profiles, we identified ten distinct cell clusters in the rat ovary. The proportion of macrophages was significantly increased in the ovaries of the OHSS model rats compared to the control group (Figure 2f). DEG analysis for each cluster showed that macrophages were the immune cell population with the highest number of DEGs (Figure 2g). Pathway enrichment analysis revealed that genes expressed higher in ovarian macrophages from OHSS rats were enriched in those whose products mediate cellular responses to stress, cytokine signaling in the immune system, and positive regulation of vasculature development. Notably, the neutrophil degranulation pathway was significantly enriched among both highly and lowly expressed genes, further indicating an overactivated immune state in OHSS, which may trigger compensatory suppressive mechanisms (Figure 2h). Furthermore, we systematically analyzed the expression patterns of key pro‐inflammatory factors (*Egf2, Gdnf, Il1a, Il1b, Il6, Tnf, Cxcl1, Jun, SerpinE1*) and anti‐inflammatory factors (*Il15, Il10, Il4, Il4r, Cxcl24*) in ovarian tissues, and found that OHSS ovaries exhibited a more pronounced inflammatory state (Figure 2i).

To further elucidate the regulatory role of ovarian macrophages in the pathogenesis of OHSS in rats, we depleted ovarian macrophages using clodrolip and evaluated the impact of macrophage ablation on OHSS phenotypic features (Figure 2j). Quantitative analysis of macrophage marker genes (*C1qa, C1qb, Mrc1*) together with CD68 immunofluorescence staining confirmed the effective depletion of ovarian macrophages (Figure 2n; Figure S3f). Compared to OHSS rats injected with the solvent control, macrophage‐depleted OHSS rats showed a further increase in ovarian volume (Figure 2j,k) and significantly higher VEGF and estradiol levels (Figure 2m). Given the known role of macrophages in regulating *corpora lutea* formation [16], their depletion partially attenuated the excessive development of *corpora lutea* induced by OHSS (Figure 2l). qRT‐PCR results further demonstrated that after macrophage ablation, the expression of genes related to inflammatory markers (*Il6, Il1b, Il1a*) was significantly upregulated (Figure 2o). This conclusion was further supported by ELISA measurements of IL6, IL1B, and IL1A levels in both serum and ovarian tissues (Figure 2p). In summary, these findings indicate that ovarian macrophage populations participate in the response to ovarian hyperstimulation by modulating local immune reactions during OHSS progression.

### 2.3 The Critical Role of Macrophages in Regulating the Ovarian Inflammatory Immune Microenvironment is Conserved in *Fshr^449A^* Knock‐in Mice

To further investigate whether macrophages represent the key cell type responsible for their evolutionary adaptation to avoid OHSS in estrildid finches, we dissected the regulatory role of macrophages using an *Fshr^449A^* knock‐in mouse model (*Fshr^449A^*). Compared to wild‐type (WT) littermates, homozygous mutant mice exhibited increased susceptibility to OHSS following stimulation with PMSG and hCG. This was evidenced by significant increases in ovary size, ovary weight, ovary‐to‐body weight ratio, and serum estradiol and VEGF levels (Figure S5a–c). scRNA‐seq analysis of ovaries from WT and mutant mice under OHSS conditions, combined with cell cluster identification using marker genes, revealed ovarian cellular composition and proportions via uniform manifold approximation and projection (UMAP) visualization (Figure S5d,g). Notably, the macrophage population was slightly expanded in *Fshr^449A^* mice (Figure S5e). Comparative analysis of the number of differentially expressed genes indicated that macrophages underwent the most extensive transcriptional changes among all immune cells (Figure S5f). Pathway enrichment analysis of macrophage DEGs demonstrated that they not only regulate the activation of various immune cells within the ovary, triggering a robust inflammatory response, but also initiate negative feedback regulatory pathways to prevent ovarian dysfunction caused by excessive immune activation (Data Figure S5h). Our quantitative results confirmed this observation: in ovaries of OHSS mice carrying the point mutation, inflammatory regulators (*Il6, Il1b, Il1a*) were significantly upregulated, while immunomodulatory genes responsive to external stimuli (*Smpdl3a, Mpeg1, Epsti1, Unc93b1, Apobec1*) were downregulated (Figure S5i,j). These results indicate that within the OHSS‐affected ovary, macrophages initiate a complex immunoregulatory network and serve as a key regulatory cell population in the pathogenesis of OHSS.

### 2.4 GPR183 represents a Promising Therapeutic Target for the Treatment of OHSS

**FIGURE 3:**
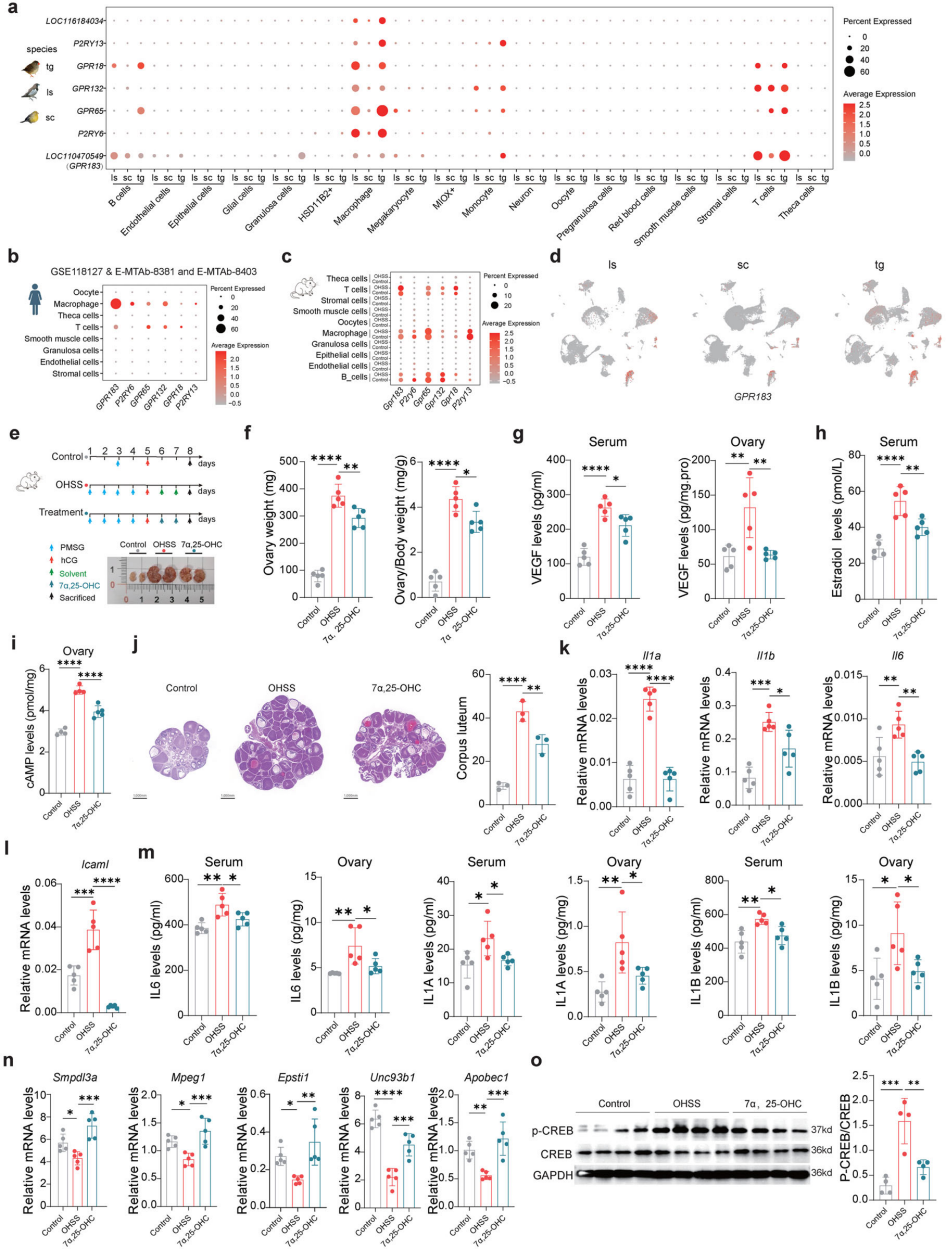
GPR183 as target for OHSS treatment (a), Expression distribution of seven differentially expressed GPCR genes derived from macrophages in the ovarian single‐cell data of estrildid and non‐estrildid finches across different cell types. (b,c), Expression distribution of the aforementioned seven differential GPCR genes in human ovarian single‐cell data (b) and in the control vs. OHSS groups in rats (c). (d), Expression distribution of *GPR183* (*LOC110470549*) in the ovarian single‐cell data of tg, ls, and sc. Red dots represent cell populations with high GPR183 expression. (e), Scheme for intraperitoneal administration of GPR183 agonist 7α,25‐OHC in OHSS rats and representative ovary images (*n* = 5). Intraperitoneal injections of 7α,25‐OHC were administered for two consecutive days, starting 24 h after hCG injection (green arrows). (f,j), Ovary weight and ovary‐to‐body weight ratio (f), serum and ovarian VEGF levels (g), serum estradiol levels (h), ovarian cAMP levels (i), and representative H&E staining histology images along with *corpora lutea* counts (j) from control, OHSS, and 7α,25‐OHC treatment groups. Scale bar is 1 mm. mRNA expression levels of inflammation‐related genes (*Il1a, Il6, Il1b*) (k) and the acute inflammation‐related adhesion factor *Icam1* (l) were quantified by qRT‐PCR. (m), ELISA was performed to assess the protein expression levels of the inflammation‐related cytokines IL1A, IL1B, and IL6 in both serum and ovarian tissues. (n), qRT‐PCR validation confirmed that immune regulation‐related genes associated with estrildid were also regulated by GPR183 in the OHSS model of 7α,25‐OHC treated rats. (o), Representative Western blot images (left) and quantitative data (right) of CREB and p‐CREB in the ovaries of the control group, OHSS group, and 7α,25‐OHC group. The silhouette in panel (b) was obtained from biorender.com. The images of the tg, ls and sc birds were adapted from the works of Peter Grima, Krayker, and Juan Emilio, respectively. All were originally published on Flickr under a CC BY‐SA 2.0 license. tg bird: https://www.flickr.com/photos/wwwpgflickrcom/1744854306/; ls bird: https://www.flickr.com/photos/krayker/2124195933/in/set‐72157603490624994/; sc bird: https://www.flickr.com/photos/juan_e/6942041699/. Data in panels (f–o) were analyzed by one‐way ANOVA. Results were presented as mean ± SEM. Statistically significant differences between groups were indicated by asterisks: ^****^ *p* < 0.0001; ^***^ *p* < 0.001; ^**^ *p* < 0.01; ^*^ *p* < 0.05; ns: *p* >0.05.

To identify and characterize commonly expressed GPCRs in estrildid ovarian macrophages that may have therapeutic potential for OHSS, we integrated and analyzed ovarian transcriptomic data from multiple species. Our initial screen identified 7 differentially expressed GPCR genes that were specifically upregulated in estrildid ovarian macrophages (Figure 3a,d). Further integration of published human ovarian single‐cell data [36, 39] and our previous single‐cell RNA sequencing analysis of OHSS rat ovaries revealed that *GPR183* was highly expressed in ovarian macrophages of both rats and humans (Figure 3b,c). Co‐immunofluorescence staining of GPR183 and CD68 further validated its expression in ovarian macrophages (Figure S6a).

To evaluate the therapeutic potential of GPR183 in OHSS, we administered its endogenous agonist, 7α,25‐dihydroxycholesterol (7α,25‐OHC), in a rat OHSS model for functional validation (Figure 3e). Compared to the OHSS‐solvent group, 7α,25‐OHC treatment significantly decreased both the absolute ovary weight and the ovary‐to‐body weight ratio (Figure 3f). ELISA results further demonstrated that 7α,25‐OHC significantly reduced VEGF and estradiol levels in both ovarian tissue and serum (Figure 3g,h). Since GPR183 is known to mediate cellular responses via the Gi signaling pathway [40], the observed decrease in ovarian cAMP levels after treatment suggested that 7α,25‐OHC may exert its therapeutic effect by inhibiting Gi signaling (Figure 3i).

To further assess the impact of 7α,25‐OHC on ovarian morphology, we performed histopathological analysis of ovarian tissues. The results showed that treatment significantly ameliorated the number of OHSS‐induced *corpora lutea* and restored the orderly arrangement of granulosa‐lutein cells (Figure 3j). Given that OHSS is accompanied by abnormal activation of intraovarian immune responses, activation of GPR183 led to significant downregulation of the pro‐inflammatory factors *Il1a, Il6*, and *Il1b* (Figure 3k), as well as a marked reduction in the acute inflammation‐related intercellular adhesion molecule intercellular adhesion molecule 1 (*Icam1*) (Figure 3l). ELISA results confirmed the expression of inflammatory factors (Figure 3m). Moreover, several immune‐related factors highly expressed in estrildid ovarian macrophages were significantly reversed after 7α,25‐OHC intervention (Figure 3n). Consistent with earlier studies [41], GPR183 also functions as a negative regulator of the cAMP/PKA/CREB signaling pathway in the ovary. Its activation significantly reduced ovarian cAMP levels (Figure 3i), leading to the inhibition of phosphorylation of its downstream effector, CREB, as demonstrated upon GPR183 activation by 7α,25‐OHC (Figure 3o). Given prior evidence indicating that GPR183 regulates immune cell migration, we investigated its role in OHSS ovaries. We found that GPR183 mediated the relocation of immune cells from the stromal region to the *corpora lutea*, where its expression was significantly elevated (Figure S6b). Furthermore, stimulation with a GPR183 agonist further enhanced its expression within the *corpora lutea*. These results suggest that during OHSS progression, GPR183 reshapes the intraovarian immune microenvironment by orchestrating the migration of immune cells. Collectively, our findings underscore the considerable therapeutic potential of targeting GPR183 in restoring ovarian immune homeostasis and alleviating OHSS pathology.

### 2.5 Macrophage Depletion Abolishes the Therapeutic Effect of the GPR183 Agonist on OHSS

**FIGURE 4:**
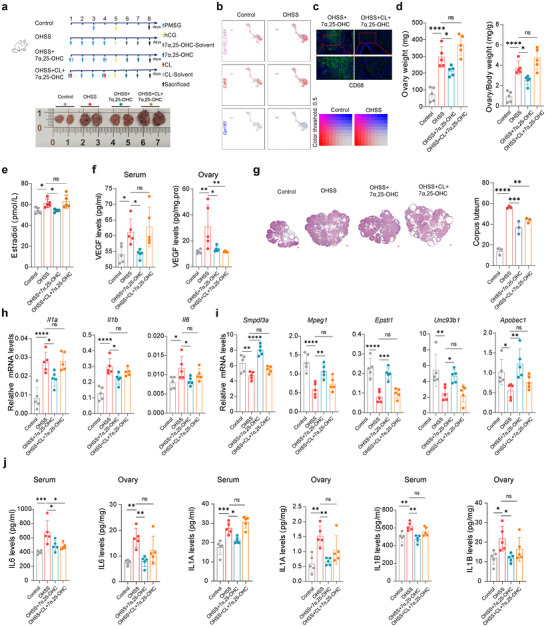
Macrophage depletion abolishes the therapeutic effect of the GPR183 agonist on OHSS (a), Schematic diagram and representative ovary images (*n* = 5) of OHSS rats treated with GPR183 agonist 7α,25‐OHC under macrophage depletion. (b), Localization and expression patterns of *Gpr183* (blue) and *Cd68* (red) in ovarian single‐cell macrophages from the control group and the OHSS group, presented as a feature fusion map (bottom right). *Gpr183_Cd68* indicates the co‐expression distribution. Darker blue represents higher *Gpr183* expression, darker red represents higher *Cd68* expression, and purple regions indicate co‐expression of the two genes through fused coloring. (c), Immunofluorescence staining of CD68 in OHSS+7α,25‐OHC group and OHSS+CL+7α,25‐OHC group in rat ovaries. The red rectangle indicates positive staining. Scale bar is 100 µm. (d–f), Ovary weight and ovary to body weight ratio (d), serum estradiol levels (e), and ovarian and serum VEGF levels (f) from control group, OHSS group, 7α,25‐OHC‐treated group (OHSS+7α,25‐OHC), and the group treated with 7α,25‐OHC after macrophage ablation (OHSS+CL+7α,25‐OHC). *n* = 5. (g), Representative H&E staining histopathological images of the maximum cross‐section of the ovaries and the number of *corpora lutea*. Scale bar is 100 µm. (h), mRNA levels of inflammation‐related genes (*Il1a, Il6, Il1b*). (i), mRNA levels of inflammation‐related genes (*Smpdl3a, Mpeg1, Epsti1, Unc93b1*, and *Apobec1*). (j), The protein levels of IL6, IL1A, and IL1B in serum and ovarian tissues were measured using ELISA. Data in panels (d–f, h–j) were analyzed by one‐way ANOVA. Data are presented as mean ± SEM. Statistical significance between groups was indicated by asterisks: ^****^ *p* < 0.0001; ^***^ *p* < 0.001; ^**^ *p* < 0.01; ^*^ *p* < 0.05; ns: *p* >0.05.

To test the hypothesis that the therapeutic effect of GPR183 is macrophage‐dependent, 7α,25‐OHC was administered to OHSS model rats after the depletion of ovarian macrophages (Figure 4a). Corroborating this approach, our prior scRNA‐seq data from control and OHSS groups indicated a strong co‐expression of GPR183 with the macrophage marker CD68 (Figure 4b), and the efficient ablation of macrophages was verified by CD68 immunofluorescence (Figure 4c). Results demonstrated that macrophage depletion prior to 7α,25‐OHC treatment abrogated the therapeutic benefits, showing no significant improvement in symptoms or in the ovary‐to‐body weight ratio compared to the OHSS group (Figure 4d). Additionally, serum estradiol and VEGF levels remained significantly elevated, with no notable difference from the OHSS group (Figure 4e,f). Pathological examination further revealed that macrophage depletion retarded *corpora lutea* development and led to a decrease in their numbers (Figure 4g). Concurrently, the expression of ovarian immune‐related pro‐inflammatory cytokines (*Il1a, Il6, Il1b*) was not effectively controlled (Figure 4h), and there was a failure to upregulate key immunoregulatory factors (Figure 4i). In addition to serum IL6 expression, ELISA results confirmed the expression of pro‐inflammatory cytokines (Figure 4j). Collectively, these data indicate that GPR183 expression on ovarian macrophages is a critical regulator in OHSS.

To further validate that the therapeutic effect of 7α,25‐OHC is specifically mediated by ovarian macrophages, we performed interventions using either intraperitoneal or intraovarian injection of clodronate liposomes (Figure S7a). Consistent with the intraperitoneal route, direct ovarian clodronate liposomes injection similarly achieved macrophage depletion and induced pathological phenotypes, including significantly enlarged ovaries and elevated VEGF and estradiol levels (Figure S7b–e). Notably, both routes of macrophage depletion effectively abolished the protective effects of 7α,25‐OHC against OHSS (Figure 4; Figure S7). These findings provide complementary evidence that the therapeutic action of GPR183 activation is critically dependent on the presence of ovarian macrophages, irrespective of the route of macrophage ablation.

### 2.6 7α,25‐OHC Modulates the Pathological Process of OHSS by Activating GPR183 and Mediating a Complex Interaction Network Among Macrophages, Endothelial Cells, Stromal Cells, Smooth Muscle Cells, and Theca Cells

**FIGURE 5:**
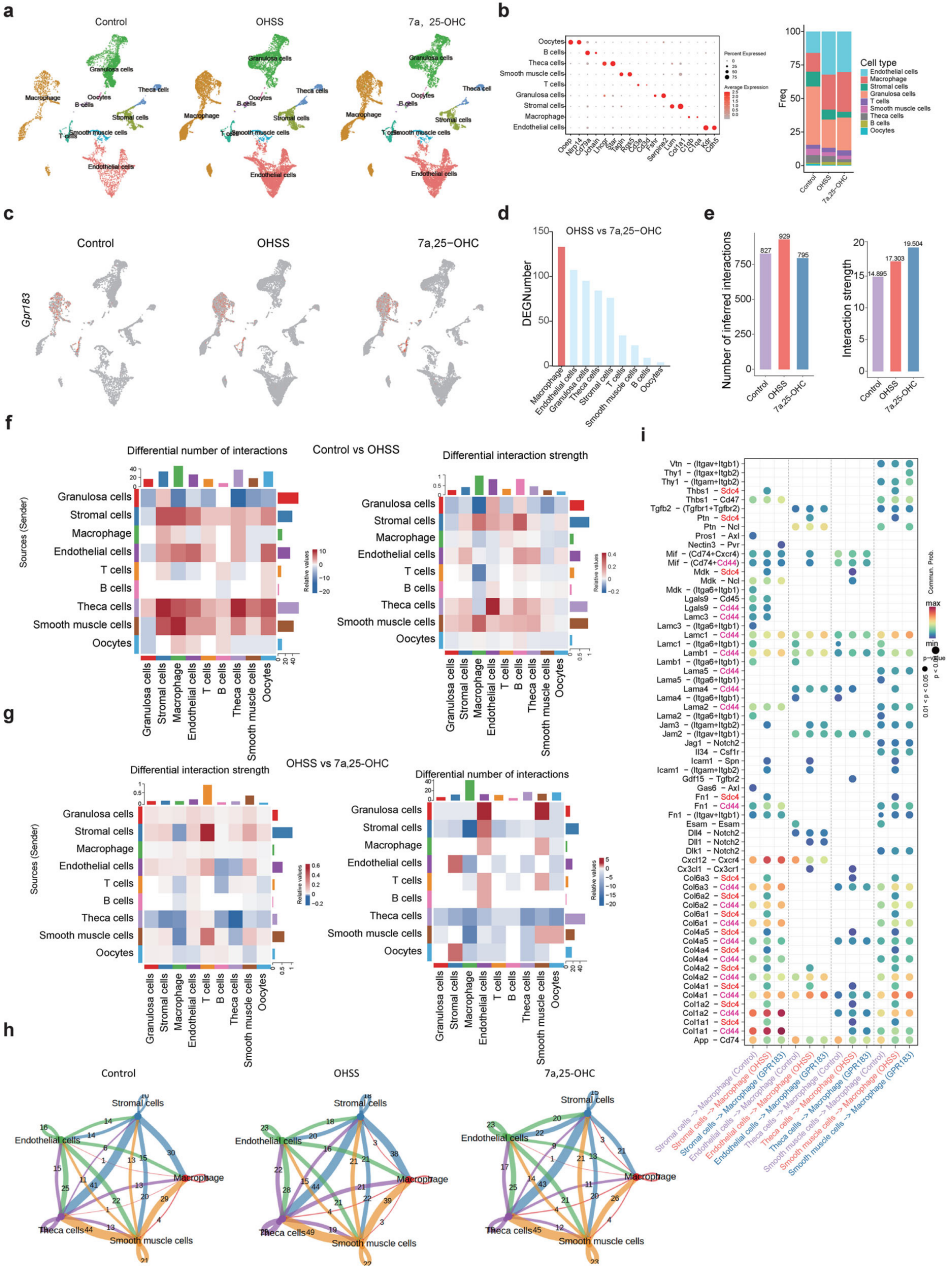
GPR183 alleviates OHSS by modulating macrophage‐mediated changes in ovarian cellular communication (a), UMAP plot of the single‐cell transcriptional atlas of rat ovaries from the control group, OHSS group, and 7α,25‐OHC treatment group (control group: *n* = 2; OHSS group: *n* = 2; 7α,25‐OHC treatment group: *n* = 2). (b), Representative genes distinguishing different cell types in the ovary (left), and proportions of each cell type (right). (c), Distribution of GPR183 in control, OHSS, and 7α,25‐OHC treatment groups. Cell populations with high GPR183 expression are highlighted by red circles. (d), Number of differentially expressed genes in each cell cluster for comparison between the OHSS group and 7α,25‐OHC treatment group. Red bar represents the number of differentially expressed genes in macrophages. (e), CellChat analysis: number and strength of total communication interactions among cell populations in control, OHSS, and 7α,25‐OHC treatment groups. (f), Number and strength of signaling interactions where each cell cluster acted as a sender and receiver, respectively, in control group compared to OHSS group. (g), Number and strength of signaling interactions where each cell cluster acted as a sender and receiver, respectively, in OHSS group compared to 7α,25‐OHC treatment group. (h), Number of mutual communications among macrophages, stromal cells, smooth muscle cells, theca cells, and endothelial cells in control, OHSS, and 7α,25‐OHC treatment groups. (i), Significantly altered ligand‐receptor pairs mediating interactions between macrophages and stromal cells, endothelial cells, theca cells, and smooth muscle cells in control, OHSS, and 7α,25‐OHC treatment groups.

To further elucidate the molecular mechanisms of GPR183 in macrophages during the pathogenesis of OHSS, we performed a systematic analysis using single‐cell data from rat ovaries treated with 7α,25‐OHC. The UMAP algorithm was applied for dimensionality reduction, and a comprehensive cell‐type atlas was constructed by integrating marker genes characteristic of each cell type in the ovary (Figure 5a). Multiple functional cell clusters were successfully identified, including granulosa cells, macrophages, T cells, smooth muscle cells, B cells, oocytes, endothelial cells, stromal cells, and theca cells. Treatment with 7α,25‐OHC led to a partial increase in the proportion of macrophages (Figure 5b). *GPR183* was predominantly expressed in macrophages and T cells, and its distribution across cell populations was not significantly altered by 7α,25‐OHC treatment (Figure 5b,c). However, this treatment induced substantial transcriptional reprogramming in macrophages (Figure 5c,d).

Pathway analysis of DEGs in macrophages revealed that, compared to the OHSS group, macrophages from the 7α,25‐OHC‐treated group showed significant upregulation of gene sets involved in biological processes such as phagocytosis, macrophage activation, and immunocyte interface regulation. Downregulated genes were primarily enriched in pathways related to atherosclerosis‐associated lipid metabolism, IL/TNF signaling, NF‐κB signaling cascade, and other inflammatory regulatory networks, while the VEGFA‐VEGFR2 signaling axis, cAMP metabolism, and steroid hormone response pathways were also markedly suppressed (Figure S8a). These functional findings suggested that activation of the GPR183 receptor modulated multiple macrophage functions, including suppression of classical inflammatory signaling such as NF‐κB, remodeling of immune microenvironment homeostasis, and regulation of hormone synthesis and vascular leakage, thereby influencing the progression of OHSS.

Given that the ovary is a highly dynamic tissue executing closely coordinated interactions among multiple cell populations during post‐ovulatory repair and regeneration, we used CellChat to analyze intercellular communications within the ovary. The results indicated that cell‐cell interactions in the OHSS group were more extensive and robust than those in the control group (Figure 5e). Although 7α,25‐OHC treatment partially enhanced the strength of specific communication pathways, it significantly reduced the number of hyperactivated ovarian cell communication links induced by OHSS (Figure 5e). To further clarify the mechanism by which GPR183‐regulated macrophages alleviate OHSS, we found that 7α,25‐OHC treatment partially reversed the OHSS‐induced alterations: specifically, it reduced the number and strength of communications received by macrophages from stromal cells, endothelial cells, theca cells, and smooth muscle cells (Figure 5f–h). At the same time, it reversed the decrease in the number of communications mediated by macrophage‐secreted factors to endothelial cells and smooth muscle cells, indicating enhanced secretory signaling function of macrophages. Further quantitative analysis revealed that in the OHSS group, interactions between the receptors SDC4 and CD44 expressed on macrophages and collagen family ligands expressed by other cell types were significantly enhanced (Figure 5i; Figure S8b). After 7α,25‐OHC treatment, SDC4‐collagen family communication was markedly weakened, while CD44‐collagen family communication was significantly strengthened, suggesting that activation of GPR183 in ovarian macrophages could regulate immune homeostasis and structural support within the ovary by modulating extracellular matrix‐related signaling, thereby maintaining tissue compartmentalization. In summary, this study demonstrated at the molecular level that 7α,25‐OHC, by activating the GPR183 receptor on macrophages, downregulated SDC4 signaling and upregulated CD44 signaling, thereby modulating the communication network between macrophages and stromal cells, smooth muscle cells, endothelial cells, and theca cells. Correction of this dysregulated intercellular signaling network was key to alleviating OHSS symptoms in rats.

## 3 Discussion

Evolutionary adaptations in non‐human vertebrates have provided a rich source of biological insight for developing novel therapeutic strategies for human diseases. From regenerative stem cells in deer antlers that repair bone defects [42] to unique porcine bile acids that improve diabetic metabolism [43], such discoveries have yielded revolutionary insights. Against this backdrop, we focused on the *FSHR* gene, which is closely associated with OHSS. Although estrildid finches carried an *FSHR^449A^* variant, in that position identical to that responsible for human disease, they did not exhibit OHSS‐like pathological features under normal physiological conditions. In vitro experiments demonstrated that this mutation led to spontaneous, elevated constitutive activity of the receptor (Figure S1a,b), conferring increased sensitivity to rhFSH, its ligand. Further in vivo experiments confirmed that even after exogenous administration of high doses of rhFSH, estrildid finches carrying a *FSHR^449A^* variant, did not develop typical OHSS symptoms. The reliability of our modeling approach was supported in mice and rats [44], and the successful induction of similar phenotypes in non‐estrildid finches.

*FSHR* is a critical gene for maintaining reproductive function in vertebrates, and its variants are closely associated with ovarian development, hormonal regulation, and fertility [45, 46, 47]. The clutch size of estrildid finches is larger than that of their sister taxon, the vidua finches [48], with the latter being obligate brood parasites of estrildid finches [49]. In response to this parasitic pressure, estrildid finches have evolved the FSHR p.Thr449Ala variant, which, although beneficial for enhancing reproductive output, may also carry an inherent risk of OHSS (unpublished observations). We propose that, over the course of evolution, estrildid finches have developed a compensatory mechanism involving ovarian macrophages that mitigates the deleterious side effects of this reproductively advantageous mutation, thereby maintaining ovarian homeostasis while preserving the benefits of enhanced fertility.

The pathological mechanism of OHSS involves an exaggerated ovarian response to ovulation‐inducing drugs, accompanied by a significant inflammatory process [50]. Elevated levels of local ovarian inflammatory factors can induce enhanced angiogenesis, increased vascular permeability, dysregulated vasodilation and constriction, and tissue edema [51]. During OHSS, inflammatory factors (such as IL6, IL1A, and TNF) in peritoneal fluid, follicular fluid, and serum are significantly elevated [52, 53].

**FIGURE 6:**
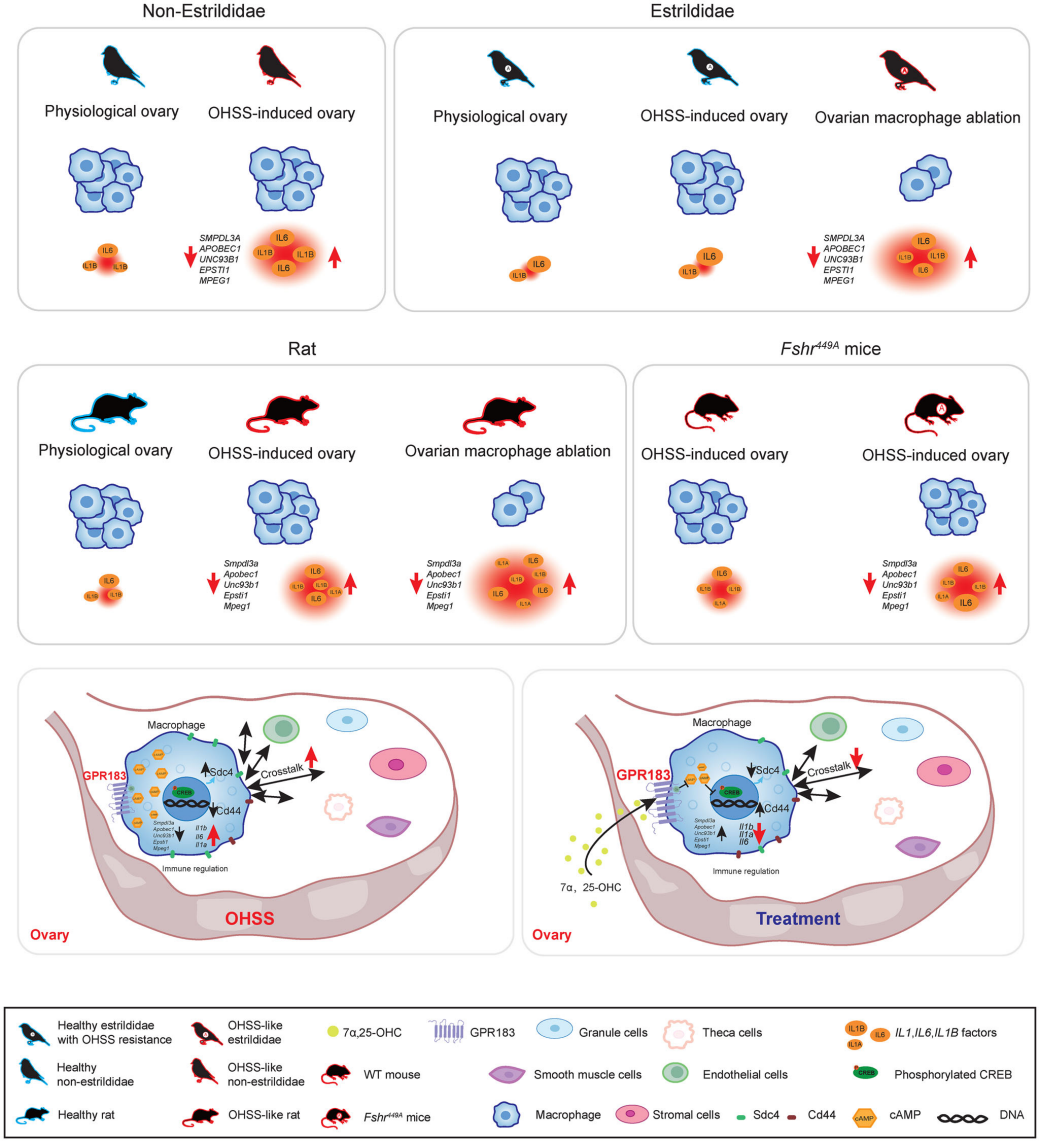
GPR183 in ovarian macrophages orchestrates the cellular communication network to regulate immune homeostasis, suggesting the targeted intervention of this axis as a novel therapeutic strategy for OHSS. Ovarian macrophages are a key immune population involved in regulating ovarian physiological processes. Depletion of macrophages in the ovarian tissue of estrildid finches reversed their natural resistance to OHSS, while depletion in rats exacerbated OHSS symptoms, a process accompanied by alterations in immune‐inflammatory factors and the involvement of GPR183 signaling. Activation of the highly expressed GPR183 receptor on macrophages effectively alleviates OHSS symptoms. The underlying mechanism involves the downregulation of pro‐inflammatory factors (such as IL1B and IL6) and the upregulation of immunomodulatory factors (such as SMPDL3A and MPEG1), thereby restoring ovarian immune homeostasis. Additionally, this signaling pathway remodels the cell‐cell communication network mediated by CD44/SDC4, leading to an improved ovarian microenvironment. This study suggests macrophages and their GPR183 receptor as potential therapeutic targets for OHSS, offering a new direction for the development of precise immune intervention strategies. Silhouettes of birds from estrildid and non‐estrildid finches were obtained from phylopic.org.

Single‐cell transcriptomic analysis revealed that ovarian macrophages in adult healthy estrildid finches undergo extensive transcriptional reprogramming and maintain a low‐inflammatory immune microenvironment (Figure 6). This finding was reciprocally validated in an *Fshr^449A^* knock‐in mouse model, where low‐dose ovulation inducing hormone stimulation elicited OHSS‐like symptoms, accompanied by dysregulated immune regulation in macrophages and elevated expression of pro‐inflammatory cytokines (Figure 6). These results collectively underscore the critical role of ovarian macrophages in maintaining immune homeostasis in estrildid finches.

To elucidate the role of macrophages in OHSS, we successfully depleted over 50% of ovarian macrophages in estrildid finches using clodronate liposomes. Combined with exogenous rhFSH stimulation, this approach effectively induced an OHSS‐like phenotype (Figure 6). Single‐cell RNA sequencing analysis of OHSS‐susceptible rats further revealed that macrophages play a crucial role in maintaining immune microenvironment stability during disease progression by regulating the balance between pro‐inflammatory and anti‐inflammatory cytokines. Consistent with this, effective macrophage depletion significantly exacerbated the severity of OHSS phenotypes in rats. Furthermore, macrophage depletion alone also resulted in significant ovarian enlargement and increased hemorrhagic spots in rats without OHSS induction (Figure S9), consistent with findings in mice [16]. These results suggest that macrophages not only participate in immune regulation during OHSS but also maintain ovarian structural integrity under physiological conditions. This loss‐of‐function study directly demonstrates that ovarian macrophages play a critical immunomodulatory role in the pathogenesis of OHSS. This study also suggested that *GPR183* might serve as a critical molecular switch in maintaining immune homeostasis within ovarian macrophages of estrildid finches. Compared to non‐estrildid species, *GPR183* expression was upregulated in ovarian macrophages of estrildid finches, while it was downregulated in ovarian macrophages of OHSS‐model rats under normal conditions. Furthermore, in vivo treatment with the endogenous GPR183 ligand 7α,25‐OHC, significantly alleviated OHSS symptoms in rats. 7α,25‐dihydroxycholesterol regulates various immune cell functions such as B cell trafficking, differentiation, and T cell‐dependent antibody responses [54]. The GPR183 receptor senses chemotactic gradients of oxysterols distributed at the borders of B and T cell zones, thereby participating in the regulation of lymphoid tissue organization [55]. Aberrant expression of GPR183 is closely associated with multiple diseases, including inflammatory or autoimmune disorders (e.g., inflammatory bowel disease [56], rheumatoid arthritis [57], type 1 diabetes, and multiple sclerosis [58], metabolic disturbances, and cancer [59], rendering it a potential therapeutic target. Although GPR183 was also expressed in other ovarian immune cells (such as T cells and B cells), depletion of ovarian macrophages markedly attenuated the therapeutic efficacy of the GPR183 agonist. Although GPR183 is also expressed in ovarian T cells, its expression in T cells alone is insufficient to mediate protection, as macrophage depletion completely abrogated the therapeutic effect of the GPR183 agonist. Following 7α,25‐OHC treatment, T cells exhibited transcriptional changes associated with immune response regulation (Figure S10), suggesting that T cells may play a supportive role, whereas macrophages serve as the primary mediators of GPR183‐dependent therapeutic effects. Future T cell‐specific knockout studies will help clarify the relative contributions of these two cell types. Additionally, while *Gpr183* is expressed in macrophages across various tissues based on public datasets (Figure S11), its expression in ovarian macrophages was dynamically regulated under OHSS conditions and functionally associated with key pathological processes such as inflammation and vascular permeability. Importantly, the therapeutic efficacy of the GPR183 agonist was attenuated not only by systemic macrophage depletion (Figure S3g) but also by local macrophage depletion via direct intraovarian injection, further supporting that the protective effect of GPR183 activation is critically dependent on ovarian‐resident macrophages rather than systemic populations.

Thus, despite carrying the pathogenic, hyperresponsive *FSHR^449A^* variant, estrildid finches maintain ovarian immune balance and reduce the incidence of OHSS by endogenously upregulating GPR183 expression in macrophages.

Further mechanistic investigation revealed that GPR183 modulates the progression of OHSS in a manner dependent on macrophage‐mediated intercellular communication (Figure 6). We found that under OHSS conditions, GPR183 treatment markedly induced transcriptional reprogramming in ovarian macrophages, enhanced macrophage‐associated signaling related to immune homeostasis, reduced the expression of inflammatory factors, and effectively restored the dysregulated cell‐cell communication network (Figure 6). At the molecular level, these effects were mediated in part through downregulation of the *SDC4* signaling pathway and concurrent upregulation of *CD44* signaling in macrophages, which collectively attenuated communication between macrophages and stromal, endothelial, smooth muscle, and theca cells. SDC4, a membrane‐associated receptor belonging to the cell surface heparan sulfate proteoglycan family, has been implicated in cancer growth, invasion, metastasis, and angiogenesis [60], and has also been identified as a potential regulator of pro‐inflammatory responses in macrophages [61]. CD44, a proteoglycan widely identified in ovarian cancer cells, binds with high affinity to hyaluronic acid in the extracellular matrix and participates in tumor cell adhesion and migration [62]. Moreover, CD44 has been shown to suppress the expression of various pro‐inflammatory mediators (such as IL1A, TNF, IL6, IL17 and CCL2) inhibit mTOR pathway activation, and modulate the function of multiple immune cells, including macrophages, dendritic cells, and T lymphocytes [63]. In summary, activation of GPR183 signaling in macrophages alleviated ovarian inflammation and improved the local immune microenvironment by coordinately regulating the expression and function of both SDC4 and CD44, thereby influencing the progression of OHSS (Figure 6). These findings not only provide new cell‐biological insights into the pathogenesis of OHSS but also establish a theoretical foundation for the development of evolution‐based therapies targeting GPR183.

Beyond stromal and endothelial cells, macrophages also interact with granulosa cells through paracrine signaling, which is essential for ovarian function [64]. In OHSS, macrophage depletion eliminates protective signals (e.g., IGF‐1) and unleashes pro‐inflammatory cytokines (IL6, IL1B), making granulosa cells vulnerable to inflammatory damage and disrupting estradiol synthesis. Conversely, GPR183 activation in macrophages downregulates CD44, potentially promoting protective crosstalk with granulosa cells via the CD44‐hyaluronic acid axis to preserve their function. These findings explain why macrophage GPR183 activation alleviates OHSS while macrophage depletion worsens the condition.

This study has several limitations. The ovarian physiological structure of estrildid finches is not fully elucidated, and interspecies differences present a significant challenge, hindering a complete understanding of the key regulatory mechanisms in the estrildid ovary‐a common impediment in studying non‐model organisms. Moreover, while OHSS is a condition unique to human reproductive medicine and may lack clinical relevance in birds and rats, this study adopts an evolutionary medicine perspective to decipher how species have evolved adaptive mechanisms to counteract FSHR hyperactivation and to translate these insights into therapeutic strategies for human OHSS. Human OHSS is an iatrogenic condition with no exact natural counterpart. However, estrildid finches naturally carry an FSHR variant at the corresponding position as the human pathogenic p.Thr449Ala mutation, yet they do not develop OHSS symptoms; instead, they have evolved a low‐inflammatory ovarian homeostatic adaptation, providing a natural disease model that circumvents the difficulty of direct observation in human samples. Based on this finding, we validated a GPR183‐targeted therapeutic strategy in rat OHSS models, showing that the endogenous ligand 7α,25‐OHC effectively alleviated OHSS symptoms, corroborating the mechanism generated by avian evolutionary adaptation. Nevertheless, the downstream regulatory mechanisms of GPR183 during OHSS induction are not fully resolved; whether key downstream effector pathways beyond SDC4 and CD44 exist requires further genetic evidence. Follow‐up studies should investigate other biological effects elicited by GPR183 and systematically evaluate the efficacy and safety of GPR183 agonists in non‐human primate models that are more closely related to humans. Future clinical sample collection is needed to further validate these findings and facilitate clinical translation.

In summary, employing a unique evolutionary medicine perspective, this study not only revealed potential evolutionary adaptations conferring OHSS resistance in estrildid finches but also successfully translated a natural evolutionary phenomenon into a therapeutically promising target, namely GPR183. Mining biomedical strategies from the evolutionary adaptations of non‐mammalian vertebrates represents a promising new pathway for drug development, offering a novel paradigm and hope for future treatments of OHSS.

## 4 Materials and Methods

### 4.1 Animals

Bengalese finches (*Lonchura striata domestica*, ls), zebra finches (*Taeniopygia guttata*, tg), and common canaries (*Serinus canaria*, sc) were obtained from a local pet bird market in Nanjing, China. Female wistar rats were provided by GemPharmatech Co., Ltd. (Jiangsu, China). The *Fshr^449A^* knock‐in mouse model was generated on a C57BL/6 background using CRISPR‑Cas9‑mediated gene editing to introduce the human p.Thr449Ala variant (corresponding to mouse position 448). Two gRNAs were designed to create double‑strand breaks, and a donor vector containing the ACT→GCT mutation was co‑injected with Cas9 mRNA into fertilized mouse eggs. Founder (F0) animals were identified by PCR and sequencing, and bred to wild‑type mice to confirm germline transmission. Homozygous mutants and wild‑type littermates used in experiments were derived from heterozygous crosses.

All experimental animals were housed under conditions of 22°C ± 2°C, 50% ± 10% humidity, and a 12 h/12 h light/dark cycle. The mice were fed with SPF‐grade, irradiation‐sterilized standard rodent diet (Xietong Bio) and had ad libitum access to sterilized water. All experimental procedures complied with the Chinese Ministry of Health “Regulations for the Administration of Laboratory Animals” (Document No. 55, 2001) and were approved by the Animal Ethics Committee of Nanjing Normal University (Approval No. IACUC‑20210233).

### 4.2 Cell Culture and Luciferase Reporter Assay

The nucleic acid sequences of the *FSHR* gene from various species were downloaded from the NCBI database. The coding sequence (CDS) of the target gene was selected and inserted into the pcDNA3.1(+) vector, with a V5/His tag fused at the C‐terminus for signal detection and gene expression experiments. Following transfection of the V5‐tagged *FSHR* into cells, the *FSHR* gene carrying the V5 tag was expressed on the cell membrane. After cell fixation and permeabilization, incubation with a V5 antibody allowed validation of FSHR expression in the transfected cells.

HEK293T cells (Fuxiang Biotechnology, Shanghai, China) were cultured in supplemented DMEM (Gibco, Cat. #C11995500BT) supplemented with 10% fetal bovine serum (FBS) (Excell, Cat. #FSP500), 1% penicillin, and 1% streptomycin (Gibco, Cat. #15140122) at 37°C with 5% CO_2_. For plasmid constitutive activity assays, cells were co‐transfected with gradient doses of receptor/CRE‐luciferase plasmids via Yeasen transfection reagent (Cat. #40802ES03), followed by V5 antibody ELISA detection and luciferase activity detection (Yeasen, Cat. #11401ES80). For rhFSH stimulation, cells were transfected with 50 ng each of receptor and reporter plasmids (pcDNA3.1 as negative control), then treated with serial rhFSH concentrations for 12 h. Luciferase activity was measured using the same kit, with all experiments performed in triplicate.

### 4.3 Establishment of OHSS Model in Rats and Birds

For rats, 3‐week‐old females received 50 IU PMSG (NSHF) daily for 4 days followed by 30 IU hCG (NSHF); controls got 10 IU PMSG on day 3, followed by 10 IU hCG 48 h later. For mice: Four‐week‐old female wild‐type mice (WT) and their KI‐mouse (*Fshr^449A^*) littermates carrying *Fshr^449A^* mutation were utilized. Mice were received 20 IU PMSG daily for 4 days followed by 1 IU hCG. Adult birds (tg, ls, and sc) were used to construct an OHSS‐like model. The experimental group received daily intraperitoneal injections of 5 IU rhFSH (Human sources of FSH) for 5 days. The control group received intraperitoneal injections of an equivalent dose of phosphate buffered saline (PBS). Serum and ovary tissues were harvested 24 h after the hCG or rhFSH injection.

### 4.4 Therapeutic Drug Treatment Plan

Upon establishment of the rat OHSS model, 24 h after the hCG injection, therapeutic agents 7α,25‐OHC (an endogenous agonist of GPR183, MCE, HY‐113962, 5 mg/kg) were administered separately via daily injections for two consecutive days. The solvent for 7α,25‐OHC was a mixture of 20% dimethyl sulfoxide (DMSO), 20% Tween‐80, and 60% normal saline, which was dissolved using low‐temperature ultrasonication. On day 8, the rats were anesthetized with tribromoethanol, blood samples were collected via orbital bleeding, and euthanasia was performed.

### 4.5 Ovarian Macrophage Depletion

Macrophage depletion models were established using adult female zebra finches and 3‐week‐old female Wistar rats. On days 1 and 4 of OHSS modeling, clodronate disodium liposomes (Yeasen, Shanghai, China) were administered intraperitoneally at a dose of 30 mg/kg. The control group received an equal volume of blank liposomes (PBS) (Yeasen, Shanghai, China). Animals were euthanized on day 6 in accordance with institutional guidelines for the ethical use of laboratory animals.

Rats were anesthetized with Avertin (2,2,2‐Tribromoethanol; T48402, Sigma–Aldrich). The dorsal hair was shaved, and the surgical area was disinfected with iodine. A sterile scalpel was used to incise the skin and peritoneum bilaterally near the spine, lateral to the hind limbs, with care taken to avoid damaging blood vessels. The ovarian fat pad was gently exteriorized using forceps to expose the ovary. A 34‐gauge needle was used to inject 80 µL (40 µL per ovary, 400 µg/rat) of clodronate disodium liposomes macrophage depletion reagent (5 mg/mL) or the corresponding control vehicle into the ovary. After injection, the ovary was carefully returned to its original position. The peritoneum and skin were sutured separately. Rats were placed in a clean, warm cage for recovery from anesthesia.

### 4.6 ELISA Determination of cAMP, VEGF and Estradiol Levels in Serum and Ovary

Serum samples were obtained from fresh, non‑hemolyzed blood after natural clotting, followed by centrifugation at 3000 rpm (4°C) for 15 min; the clear supernatant was aliquoted without disturbing the cellular layer. Ovarian tissues were rinsed with ice‑cold PBS, dissected into appropriately sized sections, and weighed. Serum estradiol and VEGF levels in rats, along with tissue cAMP and VEGF concentrations, were measured using commercial ELISA kits (Rat VEGF/Estradiol ELISA Kits, Shanghai Enzyme‑linked Biotechnology; Rat cAMP ELISA Kit, Sango Biotech, D770001). Similarly, serum and tissue estradiol levels in birds were determined using a Chicken Estradiol ELISA Kit (JONLNBIO, JL15972), all performed according to the manufacturers’ instructions.

### 4.7 ELISA of IL6, IL1A and IL1B Levels in Serum and Ovary

Commercial enzyme‐linked immunosorbent assay (ELISA) kits were used to measure the levels of IL6, IL1A, and IL1B in rat serum and tissues. The kits were purchased from Shanghai Enzyme‐linked Biotechnology Co., Ltd. (Shanghai, China), with catalog numbers ml037361 for IL1B, ml064292 for IL6, and ml107050 for IL1A. Similarly, chicken‐specific ELISA kits (IL6, catalog no. ml059839; IL1B, catalog no. ml059835; Shanghai Enzyme‐linked Biotechnology) were employed to determine IL6 and IL1B levels in avian serum and tissues. Standard curves were generated for each assay, and all procedures were performed according to the manufacturer's instructions.

### 4.8 RNA Purification, cDNA Synthesis, and RT‐qPCR

Appropriately sized tissue samples (50 mg) were homogenized in Trizol reagent (CWBio, China) on ice to extract total RNA. cDNA was synthesized using HiScript III RT SuperMix (Vazyme, China), and qPCR was performed with ChamQ Universal SYBR Master Mix (Vazyme, China) on an ABI 7500 system. Expression levels were normalized to Gapdh (rats/mice) or ACTB (birds) and analyzed via the ΔCt method. Primer sequences for qPCR are provided in Table S2.

### 4.9 Western Blot Analysis

50 mg tissue samples were homogenized at −20°C in RIPA buffer with protease inhibitors, then centrifuged at 12 000 rpm for 15 min at 4°C to collect supernatants. Protein concentrations were measured via BCA method for normalization. Supernatants mixed with 6× loading buffer were denatured at 99°C for 5 min, separated by 12% SDS‐PAGE, and transferred to pre‐activated PVDF membranes. Membranes were blocked, incubated with primary antibodies (1:1000, PBST‐diluted) at 4°C overnight, washed with TBST, and incubated with secondary antibodies for 2 h. Protein bands were visualized by ECL; membranes were stripped for re‐probing with other antibodies. The following primary antibodies were used in this study: CREB rabbit mAb (Cell Signaling Technology; Cat. #9197), P‐CREB rabbit mAb (Cell Signaling Technology; Cat. #9198), and GAPDH mouse mAb (Abclonal; Cat. #AC033). The secondary antibodies used were anti‐mouse IgG HRP conjugate (Beyotime; Cat. #A0216) and anti‐rabbit IgG HRP conjugate (Beyotime; Cat. #A0208).

### 4.10 Hematoxylin‐Eosin (H&E) Staining

Rat ovarian tissue was fixed overnight in 4% paraformaldehyde, followed by gradient dehydration with ethanol and subsequent paraffin embedding. Sections were cut at a thickness of 5 µm from the paraffin‐embedded blocks for morphological examination. Sections were stained with hematoxylin and eosin (H&E), mounted with neutral balsam, and air‐dried at room temperature. Finally, the sections were viewed under a light microscope. This study statistically analyzed the *corpora lutea* in the central region (containing blood clots due to bleeding during ovulation) and peripheral region (*corpora lutea* containing cells arranged in a string‐like pattern, rich in capillaries and connective tissue) through ovarian HE staining sections.

### 4.11 Immunofluorescence Staining

5 µm paraffin‐embedded sections (4% paraformaldehyde‐fixed) were deparaffinized and rehydrated. Antigen retrieval was conducted in sodium citrate buffer via microwave heating (medium‐high power, 25 min). Sections were washed with PBST, blocked with 5% BSA, 0.3% Triton X‐100, and incubated with primary antibodies (1:200, diluted in PBS containing 1% BSA) at 4°C overnight. After washing three times with PBST, fluorescent secondary antibodies (1:200, diluted in PBS) were applied and incubated at room temperature for 2 h, followed by another three PBST washes, the sections were stained with DAPI (Beyotime, Cat. #C1002) at room temperature for 1 min to specifically label the nuclei. Finally, the sections were mounted with an anti‐fluorescence quenching mounting medium (Beyotime, Cat. #P0126). The prepared slides were temporarily stored at 4°C and imaged using a positive fluorescence microscope. The primary antibodies used included GPR183 (Proteintech, Cat. #12377‐1‐AP) and CD68 (Abclonal, Cat. #A20803). The secondary antibody used was Goat Anti‐Rabbit IgG (H+L) Dylight 488 (Bioworld, Cat. #BS10017).

### 4.12 Multicolor Immunofluorescence Staining

Ovarian tissues were fixed in 4% paraformaldehyde for 24 h, routinely dehydrated, embedded in paraffin, and sectioned at 5 µm thickness. The sections were mounted on slides for subsequent use. A multiplex fluorescence detection kit (Immunoway, RS0037) was employed. Deparaffinization and antigen retrieval were performed simultaneously using the kit's combined deparaffinization and retrieval solution by microwave heating at high temperature for 25 min. After cooling, endogenous peroxidase activity was blocked by incubation with blocking solution for 15 min. The sections were then incubated with the primary antibody against GPR183 (1:200, diluted in PBS) for 2 h at room temperature. Following washing, the sections were incubated with HRP‐conjugated secondary antibody for 1 h at room temperature. After another wash, the tyramide‐fluorophore conjugate D‐488 solution was applied for 10 min at room temperature, followed by washing to terminate the reaction. Preheated antibody stripping solution was added, and the sections were incubated at 37°C for 10 min. This stripping step was repeated twice. After thorough washing, the sections were incubated with the second primary antibody against CD68 (1:100, diluted in PBS). In this round, the fluorophore was replaced with D‐647. Finally, after washing, the sections were mounted using a DAPI‐containing mounting medium, taking care to avoid air bubbles. Images were acquired using an automated upright fluorescence microscope.

### 4.13 Single‐Cell Library Preparation and Sequencing

Ovarian tissues were isolated from adult zebra finches (*n* = 3), common canaries (*n* = 3), Bengalese finches (*n* = 2), rats (including control group, *n* = 2; OHSS group, *n* = 2; 7α,25‐OHC group, *n* = 2), *Fshr^449A^* knock‐in mice (*n* = 1) and wild‐type mice (*n* = 2), single‐cell libraries were constructed from the bilateral ovaries of each individual mouse, without pooling samples from different individuals. Each group included five biological replicates prior to sequencing. Surrounding extraneous tissue was carefully removed from the ovaries in a petri dish containing ice‐cold PBS. A consistent dissociation protocol was applied across all species: ovarian single‐cell suspensions were prepared by digesting the tissues with 2 mg/mL collagenase Type II/IV (in a 1:1 ratio) at 37°C for 30 min. The digestate was then centrifuged at 9000 rpm for 10 s at 4°C. After removing the supernatant, the cell pellet was immediately placed on ice and resuspended in DPBS containing 0.04% bovine serum albumin (BSA, Sigma–Aldrich, CAS #: 9048‐46‐8). Cell count and viability (>80%) were assessed using Trypan Blue staining according to the manufacturer's instructions. During library preparation, quality control was performed at both intermediate and final stages, with intermediate QC conducted 3–4 times and final QC conducted twice, resulting in a total of 5–6 technical replicates. Samples that passed quality control were subsequently subjected to sequencing. scRNA‐seq libraries were constructed using the Chromium Single Cell 3' GEM, Library & Gel Bead Kit v3 (Catalog #1000121, 10× Genomics). Briefly, single‐cell suspensions were loaded onto the 10x Chromium System (10× Genomics) for Gel Bead‐In‐Emulsions (GEM) generation. cDNA synthesis was subsequently performed using the Single Cell 3’ Reagent Kit v3 (10× Genomics). The resulting cDNA libraries were amplified via PCR, fragmented, and finally sequenced on an Illumina NovaSeq 6000 platform.

### 4.14 Processing of scRNA‐Seq Data

Processed raw sequencing data using CellRanger (v7.0.1) for alignment and unique molecular identifier (UMI) counting against the following reference genomes: zebra finches (bTaeGut1.4), common canaries (cibio_Scana_2019), Bengalese finches (lonStrDom2), rat (GCF_036323735.1_GRCr8 and mRatBN7.2), and mouse (GRCm39). Ambient RNA in ovarian tissue libraries was removed using SoupX (v0.3.1) [65], and the filtered UMI count matrix was imported into R for downstream analysis. To ensure data quality, species‐specific cell quality control thresholds were applied as follows: common canaries: excluded cells with mitochondrial UMI percentage >20%, total UMI counts < 500, or >4000; zebra finches: excluded cells with mitochondrial UMI percentage >20%, total UMI counts < 200, or >7000; Bengalese finches: excluded cells with mitochondrial UMI percentage >10%, total UMI counts < 200, or >7000; rat: excluded cells with mitochondrial UMI percentage >15%, total UMI counts < 200, or >7500; mouse: excluded cells with mitochondrial UMI percentage >15%, total UMI counts < 200, or >7500. Prior to data scaling and principal component analysis (PCA), variable features were identified using Seurat [66]. Gene expression values were normalized using the NormalizeData method, and the top 2000 highly variable genes were selected for subsequent analyses. To correct for batch effects, canonical correlation analysis (CCA) was applied for dataset integration. We merged the top 2000 most variable genes from each dataset, identified integration anchors using the FindIntegrationAnchors function, and generated an integrated expression matrix via the IntegrateData function. The integrated data were then scaled and subjected to dimensionality reduction using RunPCA. Cell clustering was performed following the standard Seurat workflow, with visualization achieved through UMAP. Cluster marker genes were identified using the FindAllMarkers and FindMarkers functions with the following parameters: min.pct = 0.25, logfc.threshold = 0.25, and test.use = “Wilcox”.

### 4.15 Cross‐Species Comparative Analysis

For cross species scRNAseq comparison, one‐to‐one homologous genes shared by three species were retained for data integration of ovarian cells. We integrated all ovarian cells of each species using CCA (default parameter), and then divided them into major clusters using the FindClusters function. Use the FindAllMarkers function (min.cct = 0.25, logfc. threshold = 0.25, test. use = “Wilcox”) to identify positive DEGs in each species. For positive DEGs of common cell types in different species, we used the FindMarkers function (min.cct = 0.25, logfc. threshold = 1, test. use = “Wilcox”). The heat map and point map of DEGs were created using Seurat's Doheatmap. We performed PCA for normalized expression of each cell type for all species (pseudo count). The ligand receptor interaction was predicted by CellChat software [67]. Select significant DEGs based on the critical values of false discovery rate< 0.05 and | log2‐fold change |>1.

### 4.16 Bulk RNA‐Seq

According to the manufacturer's instructions, total RNA was extracted from zebra finch ovarian tissues using RNAiso Plus reagent, with RNA quality and integrity assessed using the Agilent Bioanalyzer 2100 system and RNA 6000 Nano Kit. A total of 60 poly(A) RNA‐seq libraries were constructed and subjected to 150 bp paired‐end sequencing on the BGISEQ DNBSEQ‐T7 platform at Novogene Bioinformatics Technology Co., Ltd (Beijing, China). Raw sequencing data were quality‐controlled using FastQC, and adapter sequences along with low‐quality reads were trimmed using Trimmomatic (v0.36). High‐quality clean reads were aligned to the reference genome using HISAT2 (v2.2.1), and the resulting BAM files were sorted with SAMtools (v1.3.1). Transcript assembly and gene expression quantification based on TPM values were performed using StringTie (v1.3.6). Differential expression analysis was carried out with the DESeq2 package, applying a significance threshold of false discovery rate < 0.05 and |log_2_ fold change| > 0.585. All related plotting and visualization were conducted using R.

### 4.17 Functional Enrichment Analysis

We performed functional enrichment analysis using Metascape [68] under default parameters. Prior to analysis, all genes were translated into human orthologs via Ensembl BioMart. The enrichment analysis utilized Homo sapiens as the target species, with all genes in the genome defined as the enrichment background and Gene Ontology Biological Process (GO‐BP) terms as the functional set. Significance thresholds were set as follows: a *p*‐value < 0.01 and annotation to a minimum of three genes.

### 4.18 Statistical Analysis

Data preprocessing was performed to ensure reliability, including data normalization and evaluation of outliers. Data are presented as the mean ± standard error of the mean (SEM). Each experiment was performed with at least three independent biological replicates (n≥3), and the sample size (n) for each analysis is indicated in the corresponding figure legends. Statistical analyses were conducted using GraphPad Prism 8.3 software. Comparisons between two groups were performed using an unpaired two‐tailed Student's *t*‐test; two‐way analysis of variance (two‐way ANOVA) was used for comparisons involving two categorical independent variables (factors), with at least two groups under each factor; and comparisons among three or more groups were carried out using one‐way ANOVA. The significance level was set at *p* = 0.05, with statistical significance defined as ^*^ *p* < 0.05, ^**^ *p* < 0.01, ^***^ *p* < 0.001, and ^****^ *p* < 0.0001; ns, not significant (*p* >0.05). The assumptions underlying the selected statistical methods were verified to be valid prior to analysis.

## Author Contributions

Conceiving and Supervising the Whole Project: C.D., S.S.Lai; Conducting Experiments: X.Y., Y.H., S.M. and J.Y.; Performing the RNA and Single‐cell Sequencing Analysis: Y.H., X.Y.; Performing data analysis and interpretation: X.Y., Y.H., S.S.Liu, L.C., B.Y., H.Z., X.H. and C.D.; Writing the Paper: X.Y., Y.H., S.S.Lai; Revising the paper: J.B., S.S.Lai, and C.D.

## Funding

This work was grants from National Natural Science Foundation of China (32170498 to S.S.Lai, 32270438 to C.D., 32570566 to C.D.); National Key R&D Program of China (2021YFF0702000 to C.D.); National Science and Technology Major Project (2023ZD0506800 to C.D.); 1.3.5 project for disciplines of excellence, West China Hospital, Sichuan University (ZYYC21011 to C.D.); National Clinical Research Center for Geriatrics, West China Hospital, Sichuan University (Z2023JC003 to C.D.); Science and Technology Department of Sichuan Province (2022YFH0116 to C.D.); the Priority Academic Program Development of Jiangsu Higher Education Institutions (PAPD to C.D.); the “Kun lun Talents” of Qinghai‐Top‐Notch and Pioneering talent of Innovation and Entrepreneurship.

## Conflicts of Interest

The authors declare no conflicts of interest.

## Supporting information




**Supporting File**: advs75523‐sup‐0001‐SuppMat.docx.

## Data Availability

The single‐cell RNA‐seq data used in this study for avian ovaries and those for Fshr449A knock‐in mouse ovaries were previously submitted to public databases, with corresponding NCBI accession numbers PRJNA1432434 and SRR37483625[69]. The newly generated single‐cell RNA‐seq data for WT mouse ovaries, single‐cell RNA‐seq data for rat ovaries, and bulk transcriptome data for avian ovaries in this study have been deposited in the NCBI database under accession numbers PRJNA1440984 (SRR37720253, SRR37720252), PRJNA1453187, and PRJNA1453241. Publicly available datasets and databases used in this study are as follows (all data under normal physiological conditions): human ovarian single‐cell (GSE118127, E‐MTAB‐8381 and E‐MTAB‐8403), rat brown adipose tissue (GSE137869), rat brain tissue (GSE137869), rat liver tissue (GSE137869), rat spleen tissue (GSM5639495), rat white adipose tissue (GSE137869), rat lung tissue (GSE273062), rat skeletal muscle tissue (GSE137869), and rat kidney tissue (GSE137869). The links to all datasets are available.
