# Supplementary material for: Natural Resistance to Ovarian Hyperstimulation Syndrome in Estrildid Finches Reveals Macrophage GPR183 as a Potential Therapeutic Target
Source: Adv Sci (Weinh). 2026 May 5;13(42):e75523. doi: 10.1002/advs.75523 (PMC13335660; doi:10.1002/advs.75523)

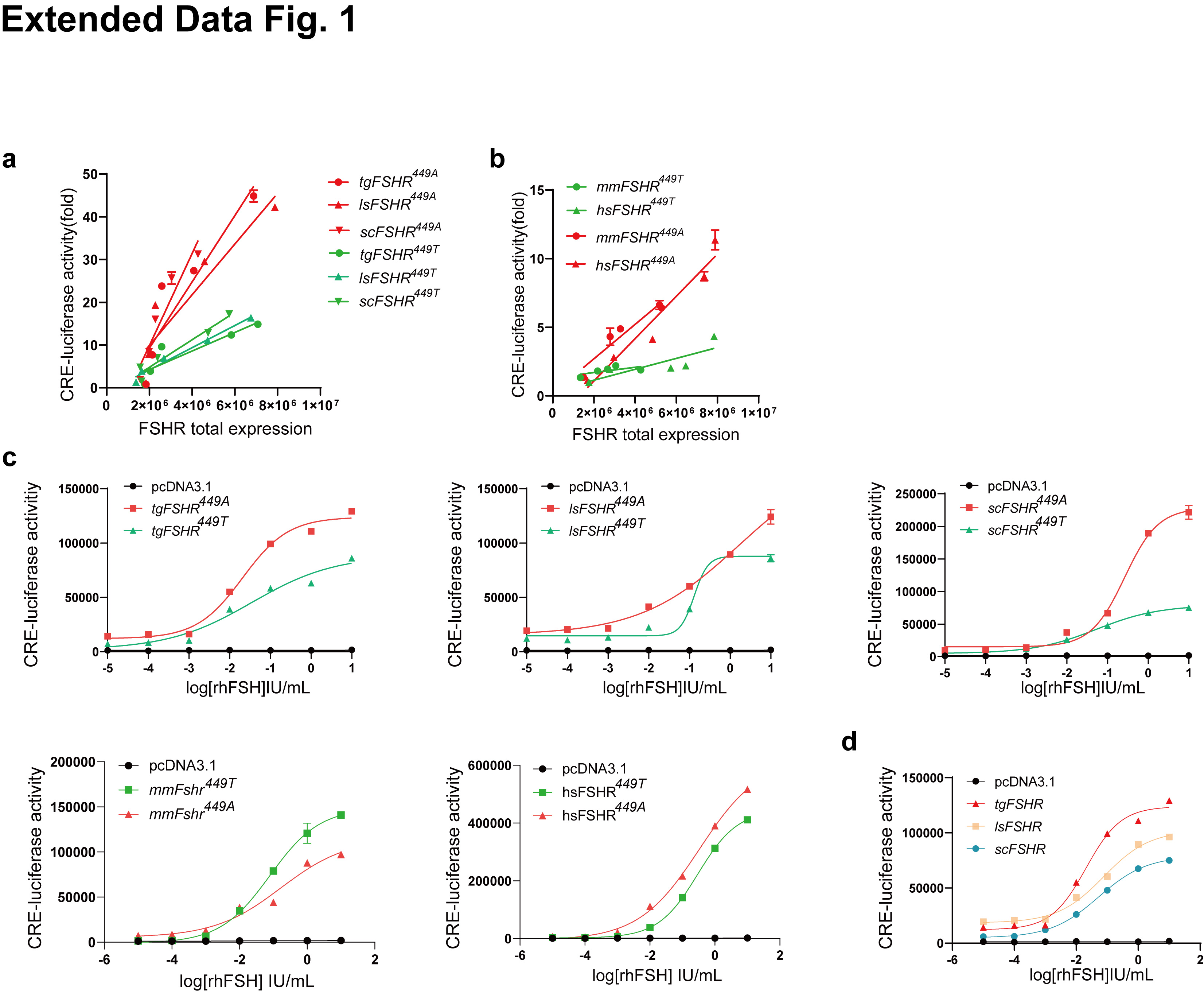


### Extended Data Fig. 1 Constitutive activity and stimulation of FSH ligands from tg, ls, and sc FSHR^449AorT^.

**a-b,** Constitutive activity of *FSHR* variants at amino acid position 449 in different species. **c,** Concentration-response curves of rhFSH (Human sources of FSH) sensitivity for tg, ls, sc, mm (*Mus musculus*) and hs (*Homo sapiens*) FSHR^449A or T^. **d,** Concentration-response curves of FSHR receptors from tg, ls, and sc in HEK293T cells following exogenous stimulation with rhFSH. Differently colored curves represent the different receptors. Refer to Extended Data Table. 1 for the matching species names with abbreviations.


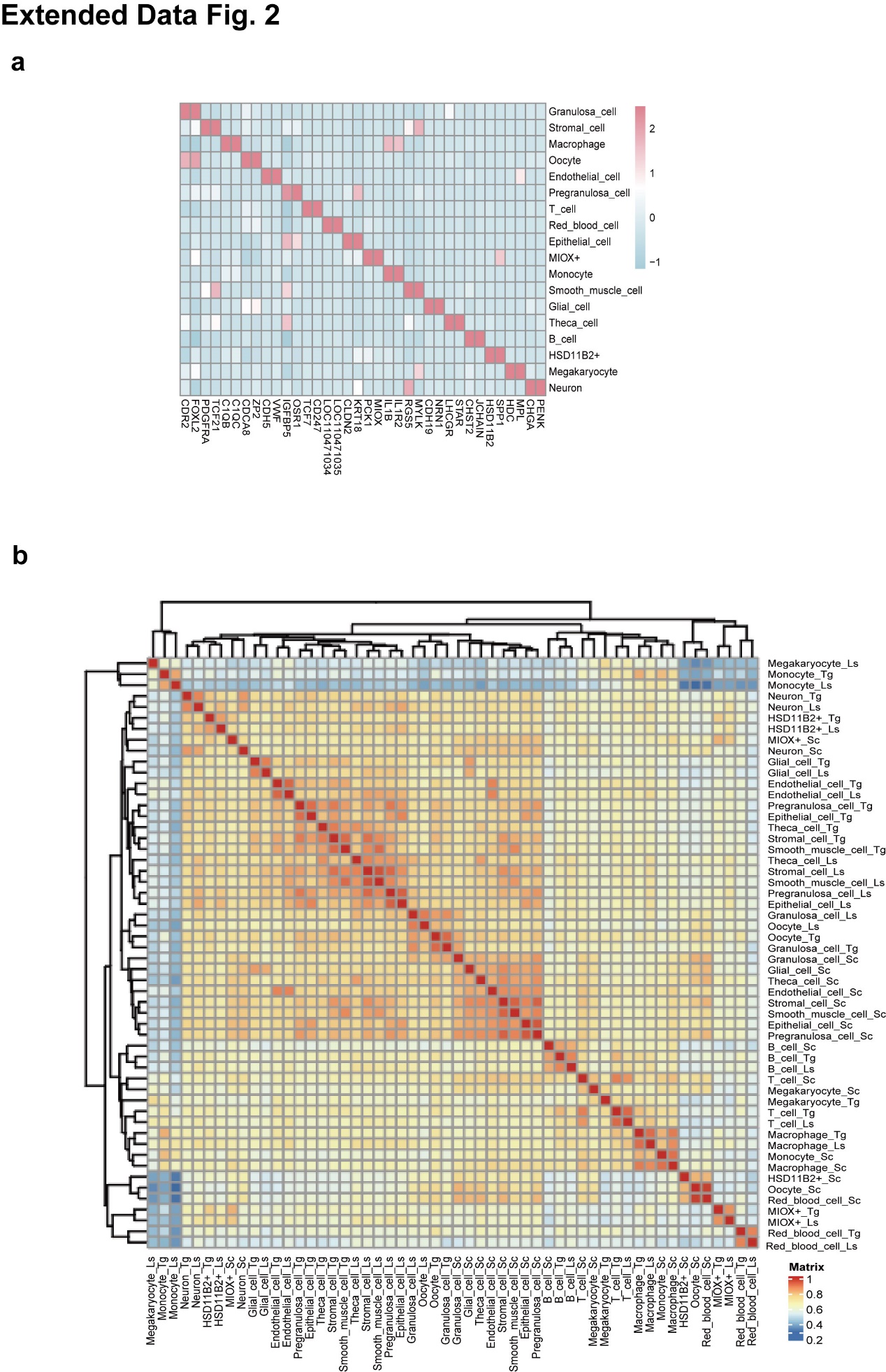


### Extended Data Fig. 2 Analysis of the estrildid and non-estrildid ovarian single-cell atlas including marker gene identification and evaluation of inter-cellular type correlations.

**a,** Heatmap displaying expression levels of canonical marker genes for 18 cell types in the ovaries of estrildid finches (tg, ls) and non-estrildid finches (sc). **b**, Heatmap reflecting correlations among different cell types across the three species (tg, ls, sc). Deeper red color indicates greater transcriptional similarity between cell types.


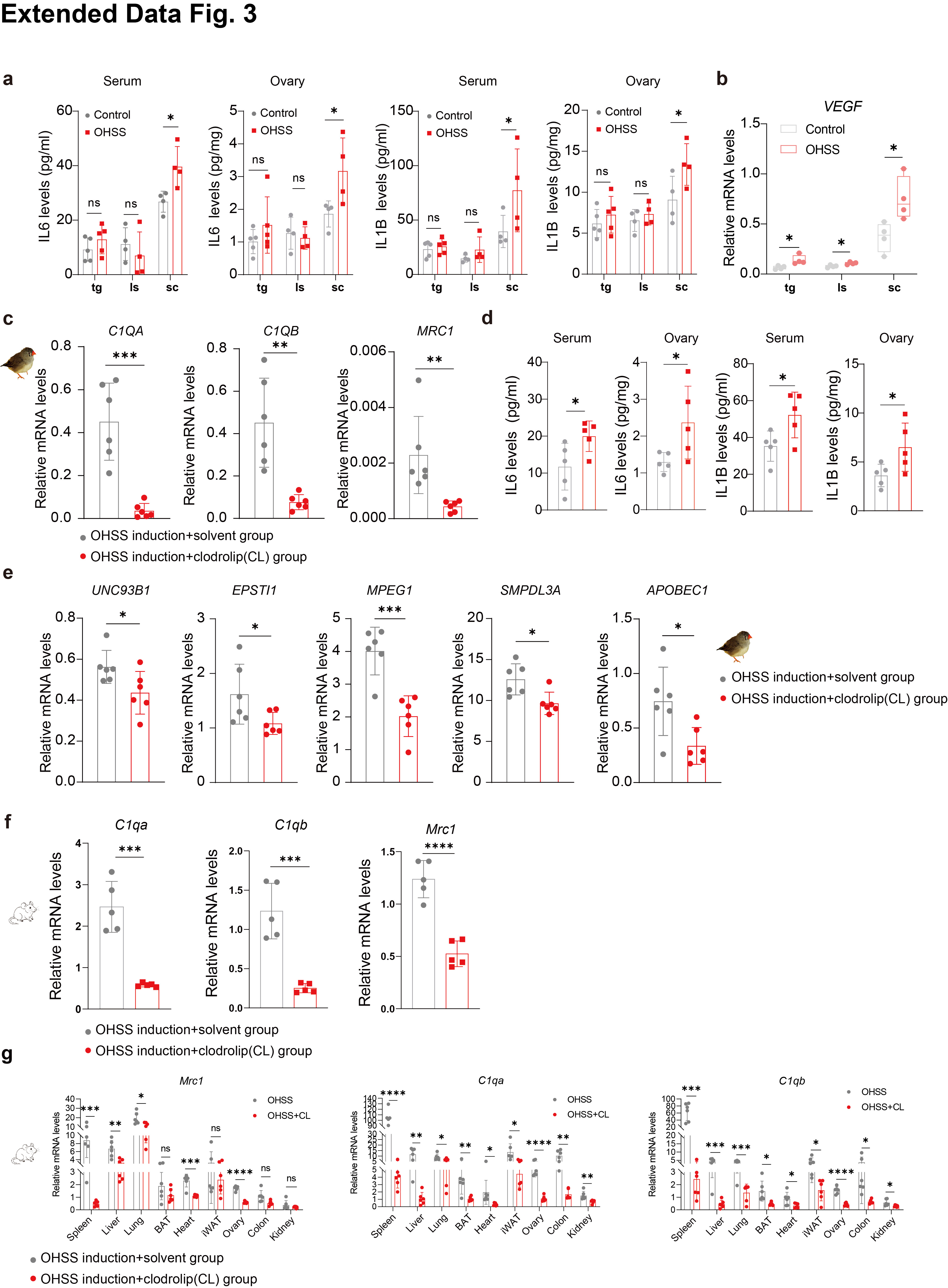


### Extended Data Fig. 3 Clodronate liposome treatment depletes ovarian macrophages.

**a,** The protein expression levels of inflammation-related factors (IL1B, and IL6) in the serum and ovarian tissues of the control group and OHSS-like group were determined in three bird species (tg, ls, and sc) using ELISA. **b,** The mRNA expression levels of the *VEGF* gene in the control and OHSS-like model groups of the three bird species were quantified by qRT-PCR. **c**, mRNA expression levels of *SMPDL3A, MPEG1, EPSTI1, UNC93B1,* and *APOBEC1* in OHSS induction + solvent group and OHSS induction + clodrolip (CL) group of *Taeniopygia guttata* (tg) under OHSS modeling conditions. **d,** The protein expression levels of inflammation-related factors (IL1A, IL1B) in the serum and ovarian tissues of the OHSS induction + solvent group and OHSS induction + CL group in tg were determined using ELISA. **e**, mRNA expression levels of macrophage marker genes (*C1QA, C1QB, MRC1*) were quantified by qRT-PCR in OHSS induction + solvent group and OHSS induction + CL group of tg under OHSS modeling conditions. **f**, mRNA expression levels of macrophage marker genes (*C1qa, C1qb, Mrc1*) were in OHSS induction + solvent group and OHSS induction + CL group of rats under OHSS modeling conditions. **g,** The RNA expression levels of *Mrc1*, *C1qa*, and *C1qb* in the spleen, liver, lung, BAT, heart, iWAT, ovary, colon, and kidney of the OHSS induction + solvent group and OHSS induction + CL group were quantified by qRT-PCR. Data in panels (**b-g**) were analyzed using unpaired t-tests; data in panel (**a**) were analyzed using two-way ANOVA. The image of the tg bird was adapted from the work of Peter Grima, originally published on Flickr under a CC BY-SA 2.0 license. Available at: https://www.flickr.com/photos/wwwpgflickrcom/1744854306/. All data are presented as mean ± SEM. Statistical significance between groups was indicated by asterisks: **P* < 0.05, ***P* < 0.01, ****P* < 0.001, *****P* < 0.0001; ns, not significant (*P* > 0.05).


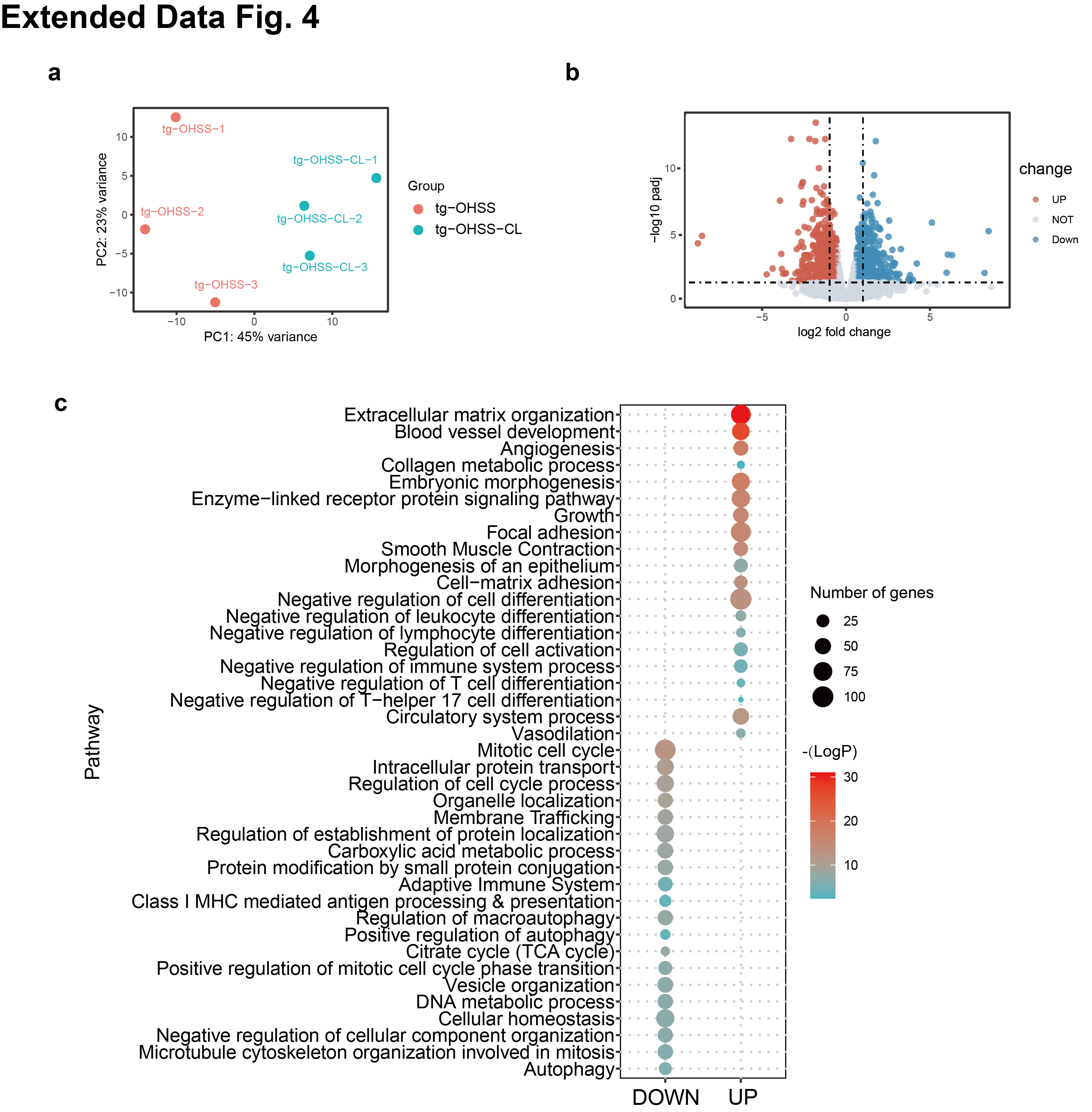


### Extended Data Fig. 4 Bulk RNA-seq analysis of ovarian tissues from *T. guttata* following macrophage ablation under OHSS conditions.

**a**, Principal component analysis plot (n = 3). **b**, Heatmap of differentially expressed genes in ovarian tissues between macrophage-ablated and control groups of the estrildid zebra finch under OHSS modeling conditions. Red indicates up-regulated genes in the macrophage-ablated group compared to controls; blue indicates down-regulated genes. **c**, Bubble plot of functional enrichment pathways for differentially expressed genes analyzed with the Metascape database.


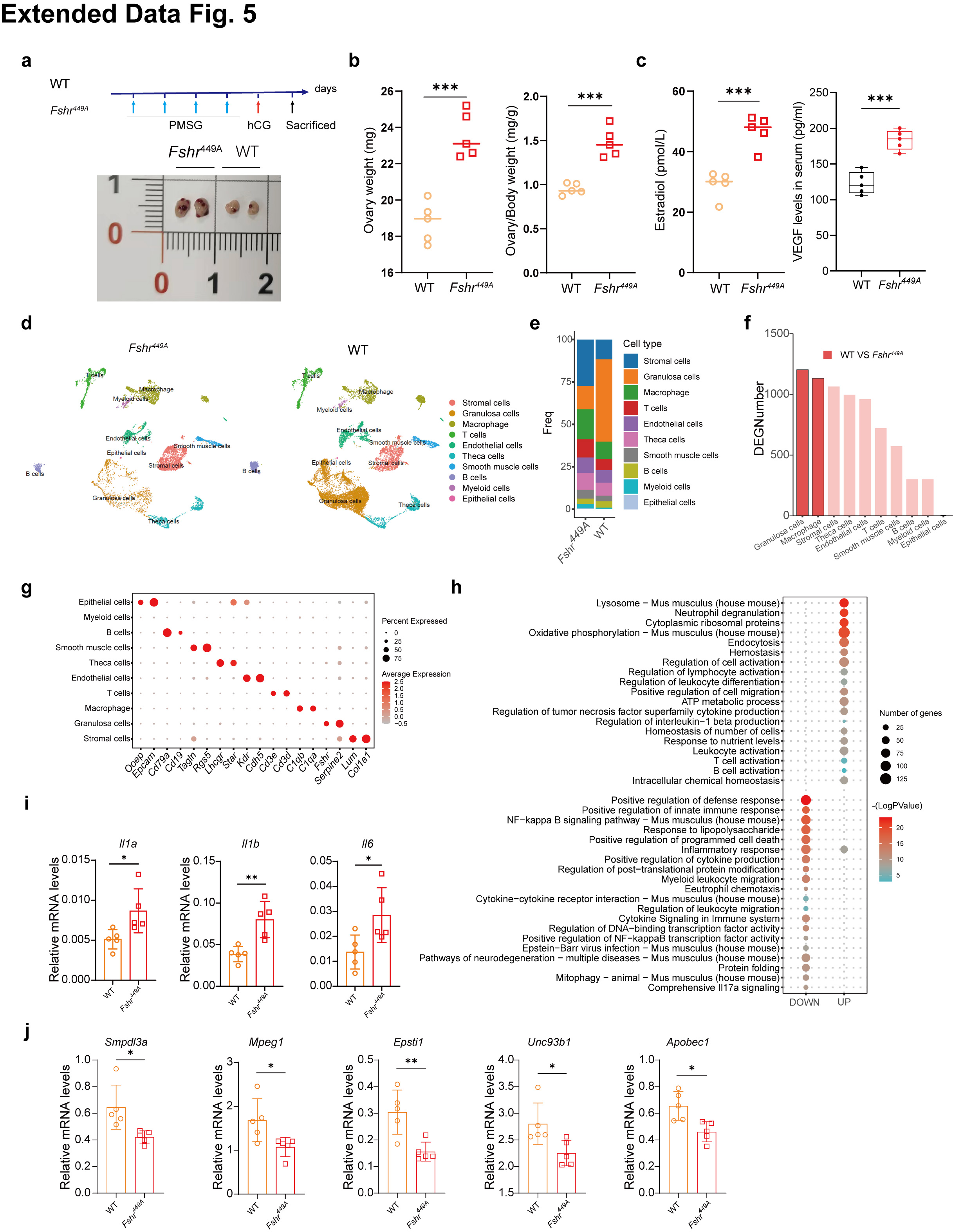


### Extended Data Fig. 5 *Fshr^449A^* increases susceptibility to OHSS-like symptoms in mice.

**a**, Scheme of OHSS-like model induction protocol in age-matched wild-type (WT) and *Fshr^449A^* homozygous mutant mice *via* intraperitoneal injection of PMSG (20 IU) and hCG (1 IU). Mice received PMSG injections for four consecutive days followed by hCG injection on day 5. Serum and ovarian tissues were collected 24 hours post-hCG administration. Representative macroscopic images of ovaries below. **b-c**, Ovary weight and ovarian/body weight ratio (n=5) (**b**), Serum estradiol and VEGF concentrations (**c**) in WT versus *Fshr^449A^* mice. **d**, UMAP projection of the single-cell transcriptomic atlas of ovarian tissues from WT and *Fshr^449A^* mice. **e**, Proportional distribution of various cell types within ovary tissue. **f**, Bar plot displaying the number of differentially expressed genes across cell types between WT and *Fshr^449A^* mice. **g**, Dot plot illustrating the expression of canonical marker genes for the ten identified ovarian cell subpopulations. **h**, Bubble plot showing pathway enrichment analysis of differentially expressed genes in macrophages from *Fshr^449A^* mice compared to WT group. **i-j**, Under OHSS modeling conditions, the RNA expression levels of *Il1a, Il6*, and *Il1b*, as well as *Smpdl3a, Mpeg1, Epsti1, Unc93b1,* and *Apobec1* in WT and *Fshr^449A^* mice. Data in panels (**b**, **c**, **i**, **j**) were analyzed using unpaired t-tests. All data are presented as mean ± SEM. Statistical significance between groups was indicated by asterisks: **P* < 0.05, ***P* < 0.01, ****P* < 0.001, *****P* < 0.0001; ns, not significant (*P* > 0.05).


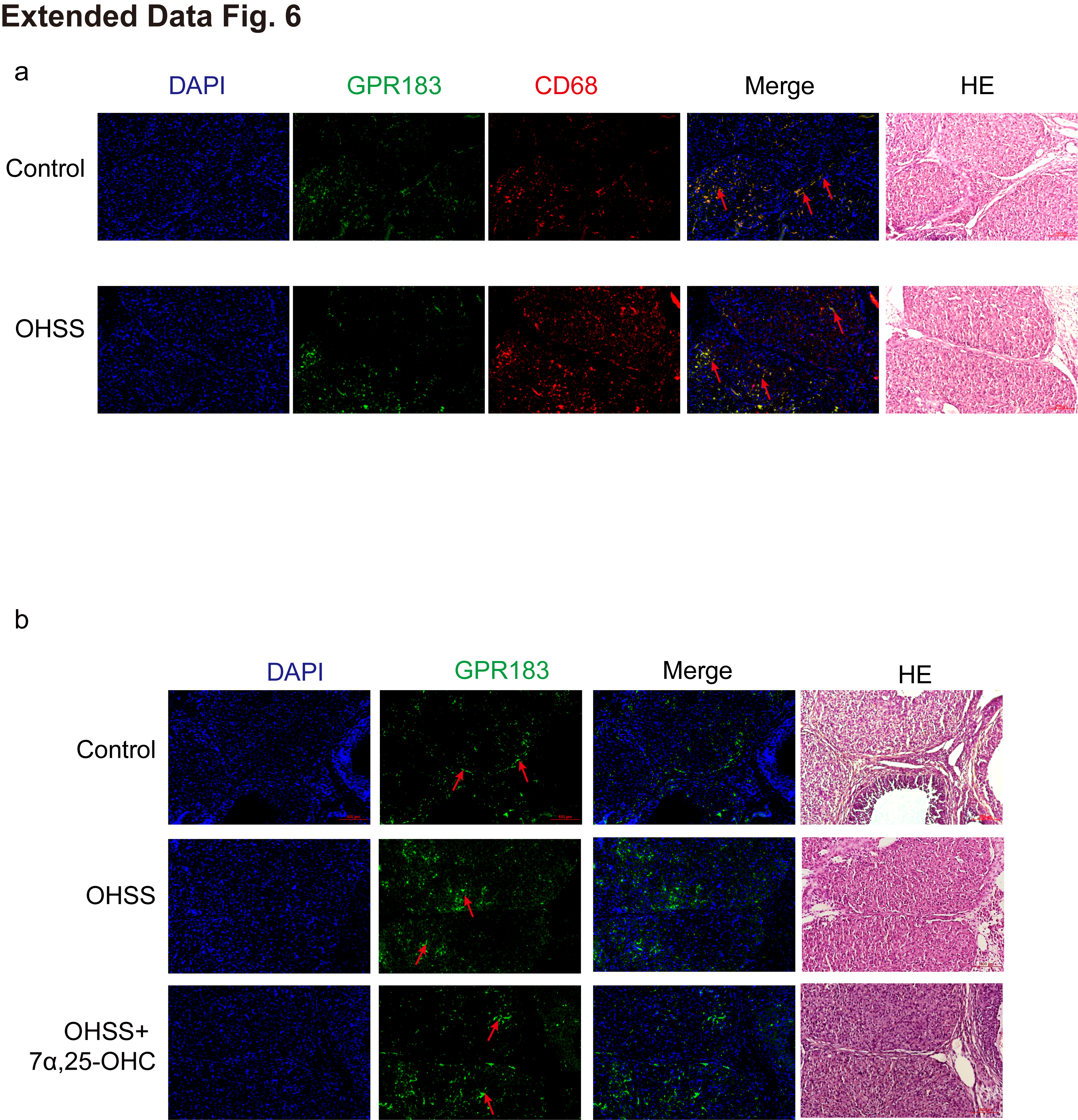


### Extended Data Fig. 6 OHSS induced the translocation of GPR183 expression from ovarian stroma to *corpora lutea*.

**a,** Immunofluorescence staining showing the localization and expression distribution of GPR183 and CD68 in ovarian tissues from the control and OHSS groups. From left to right: DAPI, GPR183, CD68, Merge, and HE images. Red arrows indicate representative regions showing specific co-localization of GPR183-positive and CD68-positive signals. Scale bar is 100μm. **b,** Localization and expression distribution of GPR183 (green) in ovarian tissues from the control, OHSS, and 7α,25-OHC treatment groups. From left to right: DAPI, GPR183, Merge, and HE images. Red arrows indicate representative regions showing specific localization of GPR183-positive signals. Scale bar =100μm.


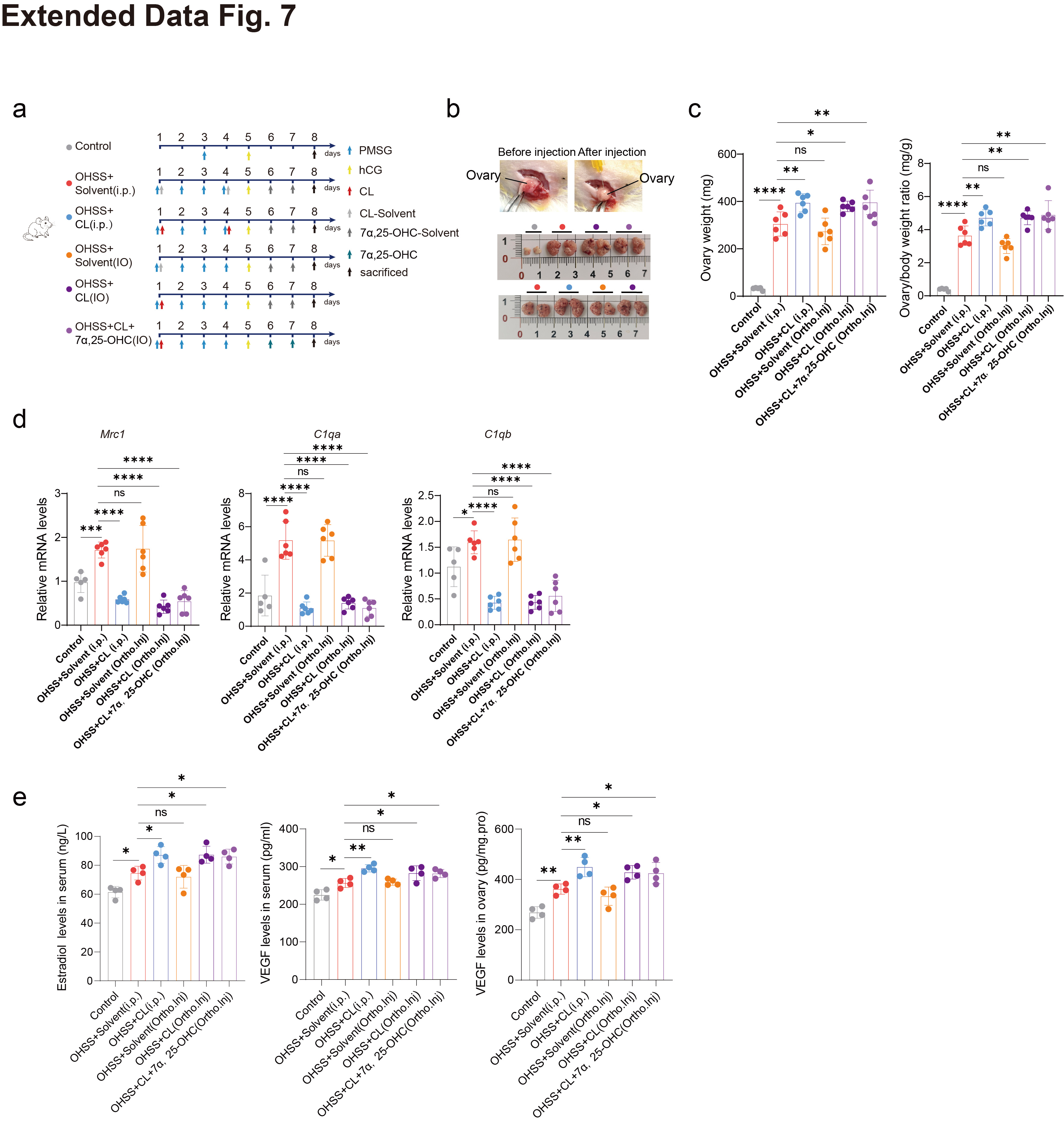


### Extended Data Fig. 7 Both intraovarian and intraperitoneal injection of macrophage depletion agents exacerbate the OHSS phenotype in rats.

**a,** The protocol for OHSS model induction and macrophage depletion agent (CL) treatment is as follows. For the intraperitoneal CL injection group (intraperitoneal injection, i.p.), 50 IU PMSG was administered intraperitoneally for four consecutive days, followed by 30 IU hCG on day 5; CL (30 mg/kg) was injected intraperitoneally on days one and four. For the orthotopic ovarian CL injection group (intraovarian injection, IO), 50 IU PMSG was administered intraperitoneally for four consecutive days, followed by 30 IU hCG on day five; CL (400 μg/rat, administered as 40 μL per ovary, total 80 μL) was injected orthotopically into the ovaries on day one. Corresponding solvent control groups received the macrophage depletion agent solvent via either intraperitoneal injection or orthotopic ovarian injection. The control group received 10 IU PMSG intraperitoneally on day three and 10 IU hCG intraperitoneally on day five. Ovaries and serum samples were collected from each group 24 hours after hCG injection (n = 6). **b,** Schematic diagram of orthotopic ovarian injection (top) and representative gross images of ovaries from each group (bottom). **c,** Ovarian weight and ovary-to-body weight ratio in each group of rats. **d,** mRNA expression levels of macrophage marker genes (*Mrc1*, *C1qa*, *C1qb*) in the ovaries of mice from each group, as determined by qRT-PCR. **e,** Serum estradiol levels, as well as VEGF concentrations in serum and ovarian tissues, in each group of rats. Data in panels (**c**-**e**) were analyzed using an unpaired one-way test. All data are presented as mean ± SEM. Statistical significance between groups is indicated by asterisks: **P* < 0.05, ***P* < 0.01, ****P* < 0.001, *****P* < 0.0001; ns, not significant (*P* > 0.05).

**
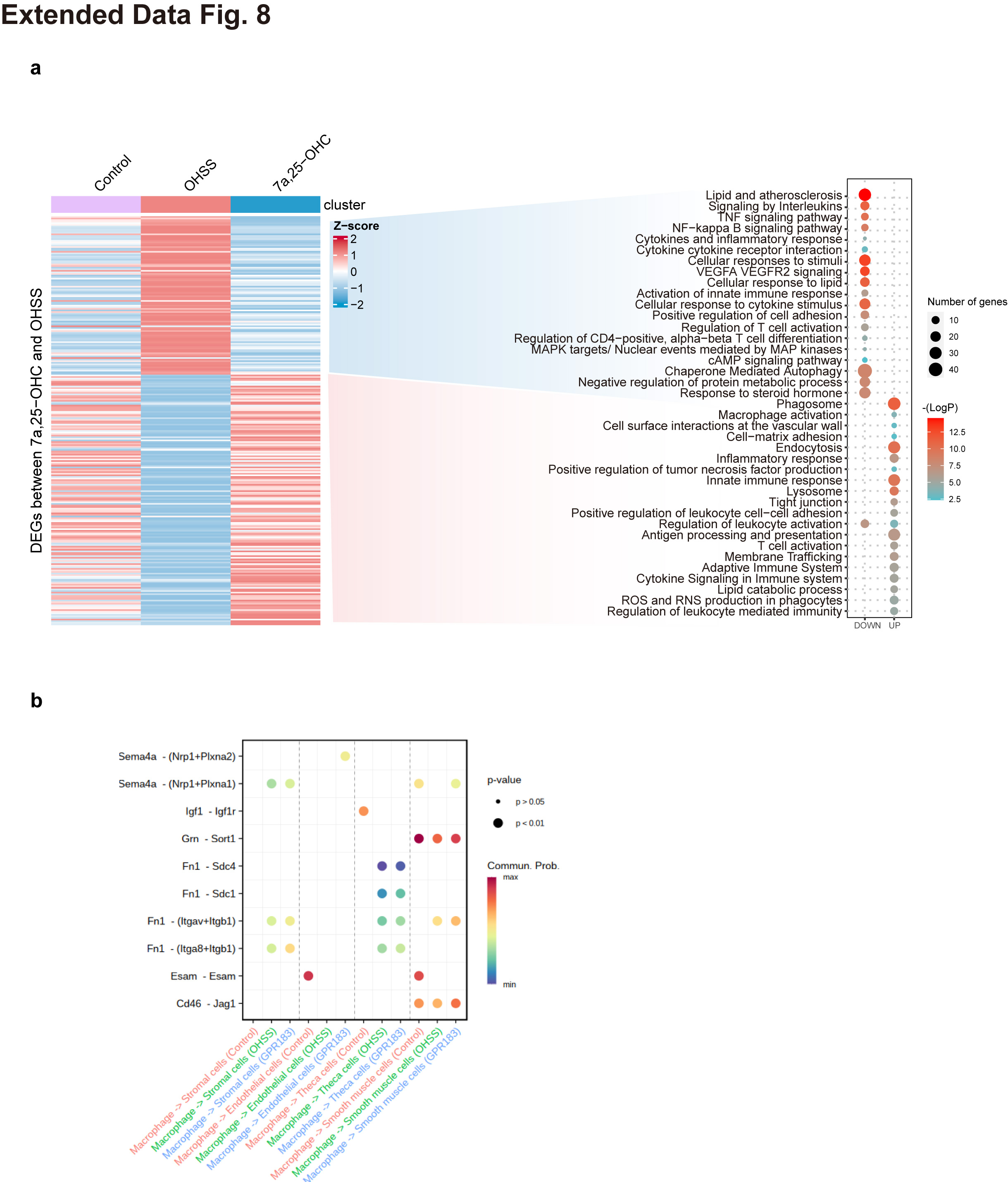
**

### Extended Data Fig. 8 Single-cell analysis of differential gene pathways and cell-cell communication following 7α,25-OHC treatment in OHSS

**a**, Pathway enrichment analysis of differentially expressed genes in ovarian macrophages from control, OHSS, and 7α,25-OHC treatment groups. Blue represents down-regulated genes after 7α,25-OHC treatment, while red represents up-regulated genes. **b**, CellChat analysis for critical ligand-receptor interactions where macrophages act as ligand senders and stromal/endothelial/theca/smooth muscle cells as signal receivers.


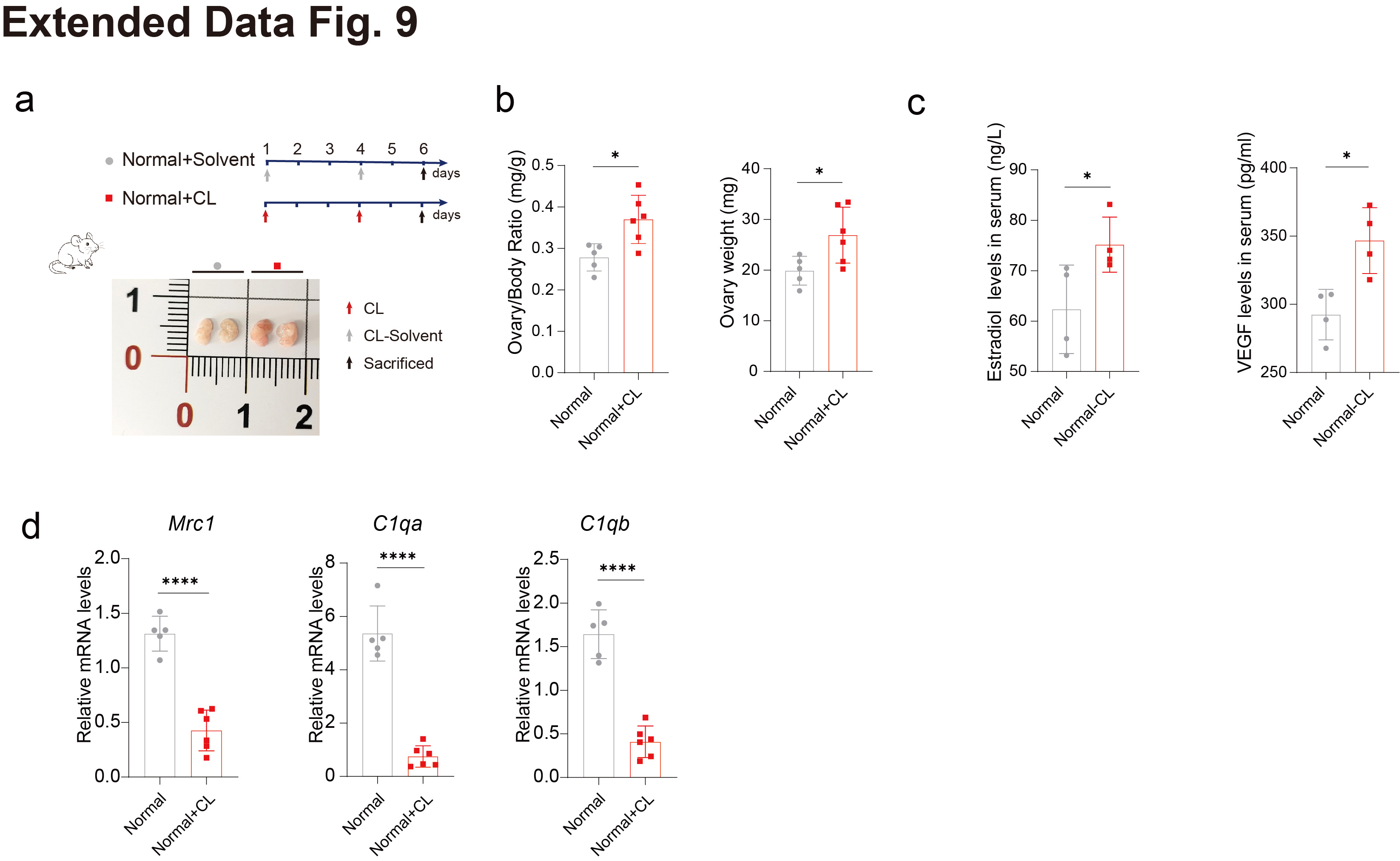


**Extended Data Fig. 9 Macrophage depletion in the ovaries leads to increased ovarian weight and elevated serum estradiol and VEGF levels in normal rats**

**a**, Experimental protocol for macrophage depletion in rats under normal physiological conditions and representative ovarian images. The macrophage depletion group received injections of the macrophage depletion agent (CL) on days one and four, while the solvent group received the solvent at the corresponding time points. Ovaries and serum samples were collected on day six (Normal group：n = 5；Normal + CL group：n = 6). **b**, Ovarian weight and ovary-to-body weight ratio in the solvent group and macrophage depletion group. **c**, Serum estradiol and VEGF levels in the solvent group and macrophage depletion group. **d**, Expression levels of macrophage marker genes (*Mrc1*, *C1qa*, *C1qb*) in the ovaries of the solvent group and macrophage depletion group. Data in panels (**b**-**d**) were analyzed using unpaired t-tests. All data are presented as mean ± SEM. Statistical significance between groups is indicated by asterisks: **P* < 0.05, ***P* < 0.01, ****P* < 0.001, *****P* < 0.0001; ns, not significant (*P* > 0.05).

**
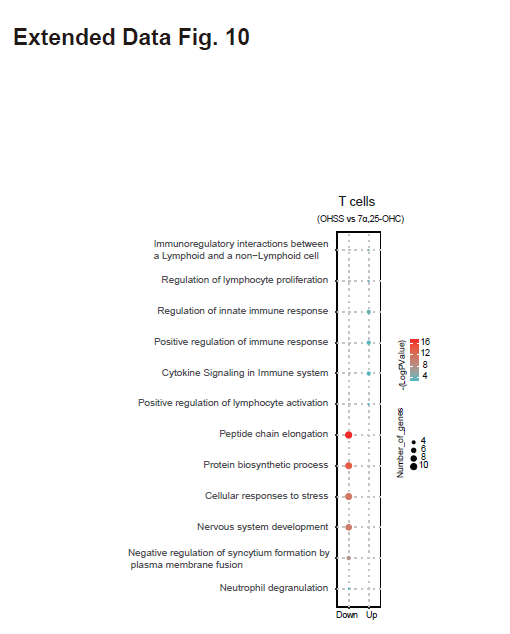
**

**Extended Data Fig. 10 Analysis of differentially expressed genes in T Cells in the ovaries of rats with OHSS following GPR183 agonist treatment**

Functional pathway enrichment analysis of differentially expressed genes in T cells from single-cell ovarian samples of the OHSS group and the GPR183 agonist (7α,25-OHC) treatment group.


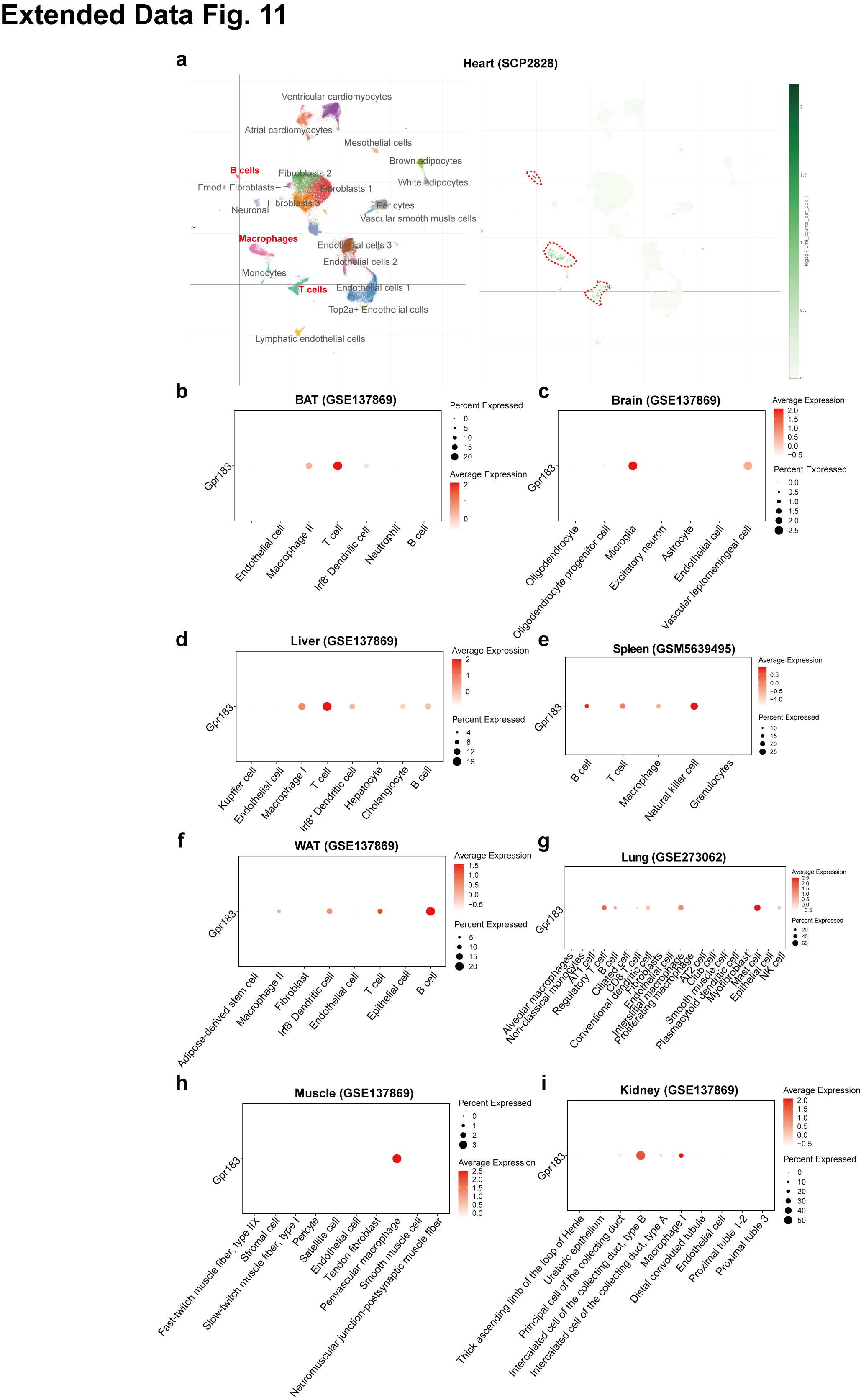


**Extended Data Fig. 11 GPR183 expression in representative tissues of rats.**

**a**, UMAP visualization of the rat heart single-cell transcriptome (dataset SCP2828), with major cell types annotated. The color gradient represents the log-transformed expression level of *Gpr183*, highlighting its enrichment in immune cell populations (B cells, macrophages, T cells, annotated in green). **b–i**, Dot plots showing the average expression levels (color intensity, red) and the proportion of expressing cells (dot size) of *Gpr183* across different cell populations in various rat tissues: Brown adipose tissue (**b**, BAT, dataset GSE137869), Brain (**c**, dataset GSE137869), Liver (**d**, dataset GSE137869), Spleen (**e**, dataset GSM5639495), White adipose tissue (**f**, WAT, dataset GSE137869), Lung (**g**, dataset GSE273062), Skeletal muscle (**h**, dataset GSE137869), Kidney (**i**, dataset GSE137869). Across all examined tissues, *Gpr183* is primarily expressed in immune cell subsets (e.g., T cells, B cells, macrophages) as well as in certain parenchymal cell types, suggesting a conserved immune-enriched expression pattern.

Extended Data Table. 1 Abbreviations information used in this study.


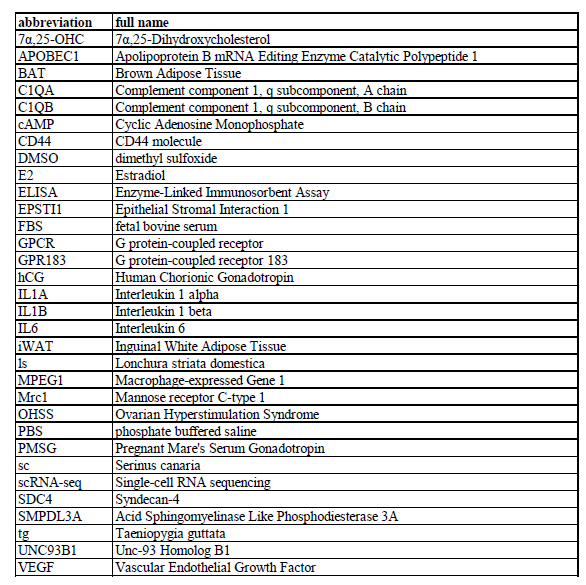


Extended Data Table. 2 Information for primers used in this study.


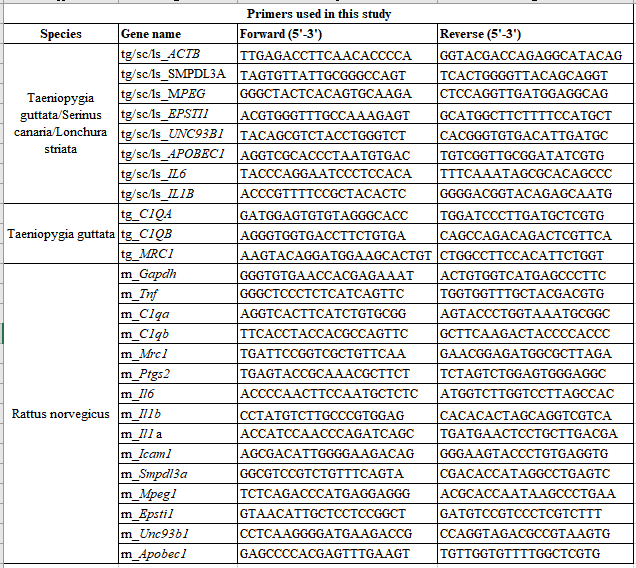


Extended Data Table. 3 Information on the amino acid at position 449 of the FSHR sequence in vertebrates.


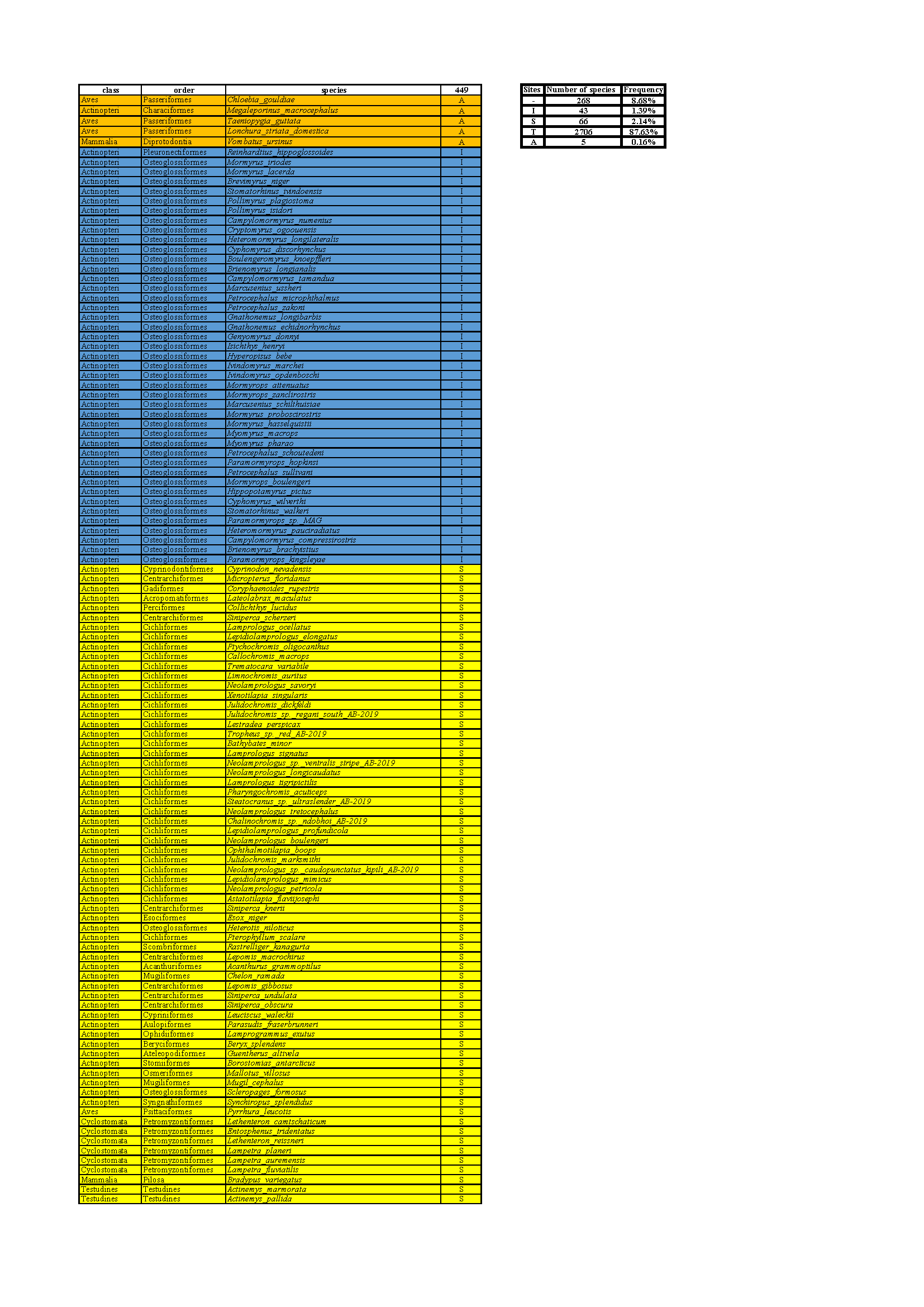

Supplement: Supplementary file 1 — Supporting File: advs75523‐sup‐0001‐SuppMat.docx. [file ADVS-13-e75523-s001.docx]
